# I-NOWJ: adaptive context selection and knowledge-guided learning for substance use named entity recognition

**DOI:** 10.1093/database/baag056

**Published:** 2026-09-25

**Authors:** Quoc-Viet-Anh Tran, Gia-Bao Duong, Huu-Huy-Hoang Tran, Thi-Hai-Yen Vuong, Hoang-Quynh Le

**Affiliations:** Knowledge Technology Laboratory, Faculty of Information Technology, VNU University of Engineering and Technology, 144 Xuan Thuy, Cau Giay, Hanoi 10000, Vietnam; Knowledge Technology Laboratory, Faculty of Information Technology, VNU University of Engineering and Technology, 144 Xuan Thuy, Cau Giay, Hanoi 10000, Vietnam; Knowledge Technology Laboratory, Faculty of Information Technology, VNU University of Engineering and Technology, 144 Xuan Thuy, Cau Giay, Hanoi 10000, Vietnam; Knowledge Technology Laboratory, Faculty of Information Technology, VNU University of Engineering and Technology, 144 Xuan Thuy, Cau Giay, Hanoi 10000, Vietnam; Knowledge Technology Laboratory, Faculty of Information Technology, VNU University of Engineering and Technology, 144 Xuan Thuy, Cau Giay, Hanoi 10000, Vietnam

## Abstract

Substance use information in clinical narratives is clinically important but is typically documented in unstructured and informal text, making automatic extraction challenging, particularly in low-resource and non-English settings. The BioCreative IX ToxHabits shared task addresses this problem in Spanish clinical text by identifying substance use triggers and their arguments. In this paper, we introduce I-NOWJ, which addresses these challenges through two complementary strategies: a low-resource strategy based on domain knowledge expansion, controlled data augmentation with quality-aware self-training, and an adaptive context selection mechanism that determines the appropriate contextual scope for each instance. Experimental results on the ToxHabits benchmark show that I-NOWJ achieves an F1 score of 94.93% on trigger detection and 91.30% on argument extraction. These results demonstrate the effectiveness of knowledge-guided modelling and adaptive context selection for robust clinical named entity recognition in low-resource settings. Our implementation and resources are publicly available at: https://github.com/candleMind/toxhabit

## Graphical Abstract

**Graphical Abstract:**
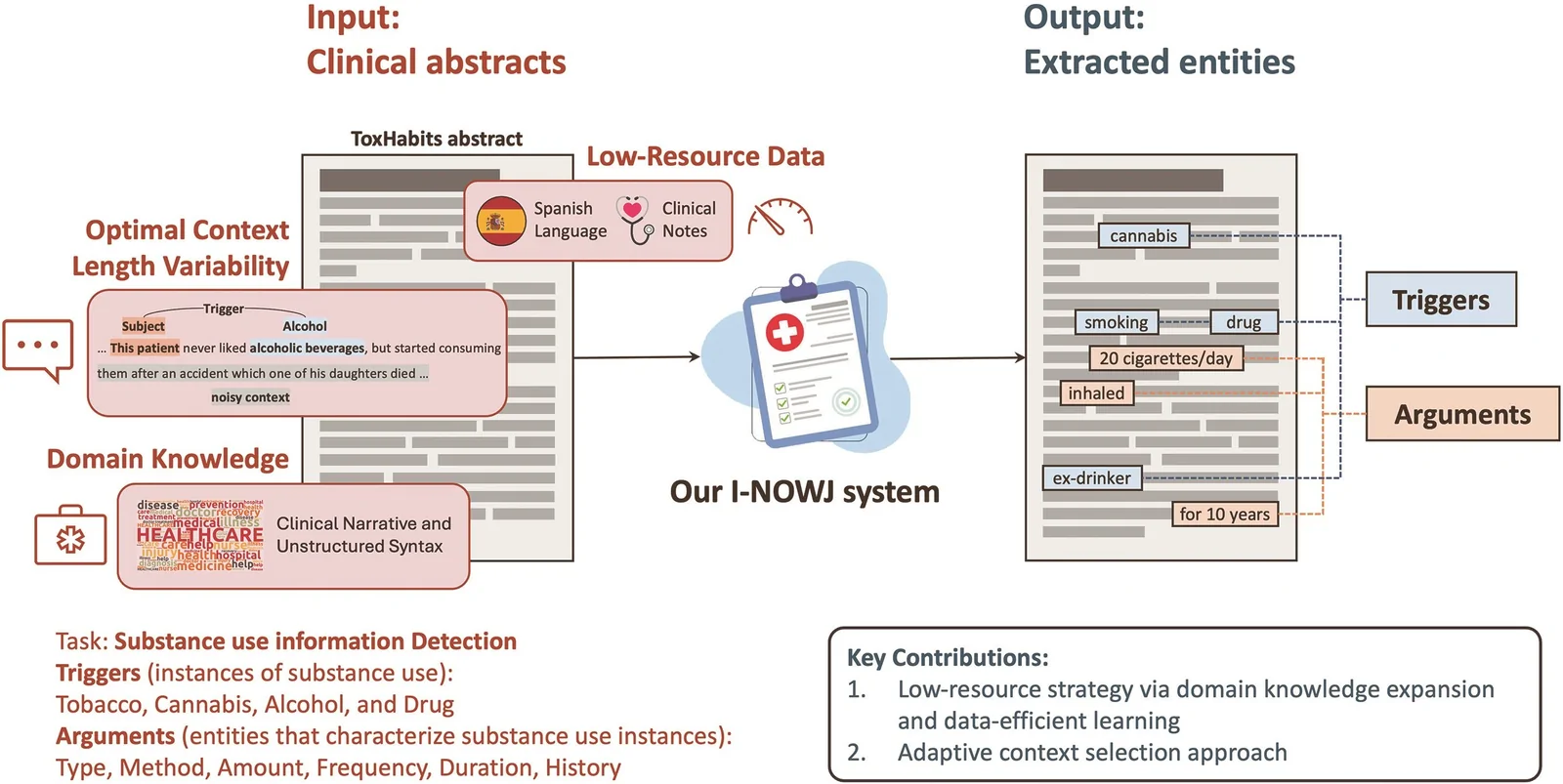

## Introduction

Named entity recognition (NER) is a core information extraction task in clinical natural language processing (NLP), converting narrative documentation into useful structured information [1]. Reliable NER systems support a wide range of downstream applications, including cohort identification and clinical outcome analysis. Substance use represents a clinically significant domain due to its strong associations with chronic disease progression, mental health conditions, and a substantial public health burden [2]. Unlike diagnoses, laboratory measurements, or medication orders that are typically captured in structured fields, substance use information is often documented only in free-text clinical narratives, including case reports, discharge summaries, and progress notes [3], making automated extraction particularly challenging. To advance research in this area, the *ToxHabits* shared task [4] was introduced to promote automatic extraction of substance use information from Spanish clinical text. The challenge comprises two subtasks: ToxNER, which identifies substance use mentions (*triggers*), and ToxUse, which extracts usage characteristics (*arguments*), such as frequency and duration (see the *Graphical abstract*). We further identify two principal groups of challenges underlying this task, as described below.

**Lexical and semantic challenges**: Although triggers and arguments are detected as independent entities, arguments are defined in relation to substance use mentions, introducing dependencies that are not typically modelled in traditional clinical NER.

Furthermore, substance use mentions frequently involve colloquial expressions, regional variants, and street-level drug slang (e.g. *meow meow* and *krokodil*), which are highly localized and largely absent from biomedical corpora. The annotation scheme also includes boundary cases between everyday products and psychoactive substances (e.g. *redbull* and *café*), as well as broad pharmacological categories rather than specific molecules, such as *hipnóticos* (‘hypnotics’) and *psicofármacos* (‘psychotropic medications’). In addition, verbs denoting consumption, such as *consumo* (‘consumption’), and noun phrases referring to individuals, such as *bebedor* (‘drinker’), require models to disambiguate between substances, actions, and agents within the same semantic space.

Argument expressions add further complexity, as they often encode numerical, temporal, and abbreviated information, including dosage, frequency, and duration.

Most existing approaches to clinical NER rely primarily on supervised learning [1, 5, 6], which limits generalization in low-resource settings where many triggers and arguments occur in the long tail and are sparsely represented in annotated data. Although external knowledge bases are widely used in clinical NLP, they are typically organized around standardized vocabularies and often derived from high-resource languages such as English [7–10]. In Spanish, biomedical NLP resources have been developed for multiple domains, including pharmacological entity recognition (e.g. PharmaCoNER [11]), diseases, symptoms, procedures, medications, and other clinical entities [12], and clinical information contained in referral narratives [13]. However, resources specifically addressing toxic habits and substance use remain scarce. As a result, both supervised and knowledge-based methods struggle to capture informal and semantically nuanced substance use expressions in Spanish clinical notes. These observations motivate our *low-resource strategies* that do not rely solely on lexicon expansion or indiscriminate data augmentation. Effective modelling requires knowledge-aware representations that leverage richer linguistic resources available in English while remaining robust to informal and non-standard usage. In addition, data-efficient learning mechanisms are needed to improve generalization without introducing noise. Guided by this motivation, we pursue two approaches tailored to low-resource settings: expanding domain knowledge through carefully constructed dictionaries of formal and informal terms, and enhancing robustness through controlled data augmentation combined with quality-aware self-training.

**Figure 1:**
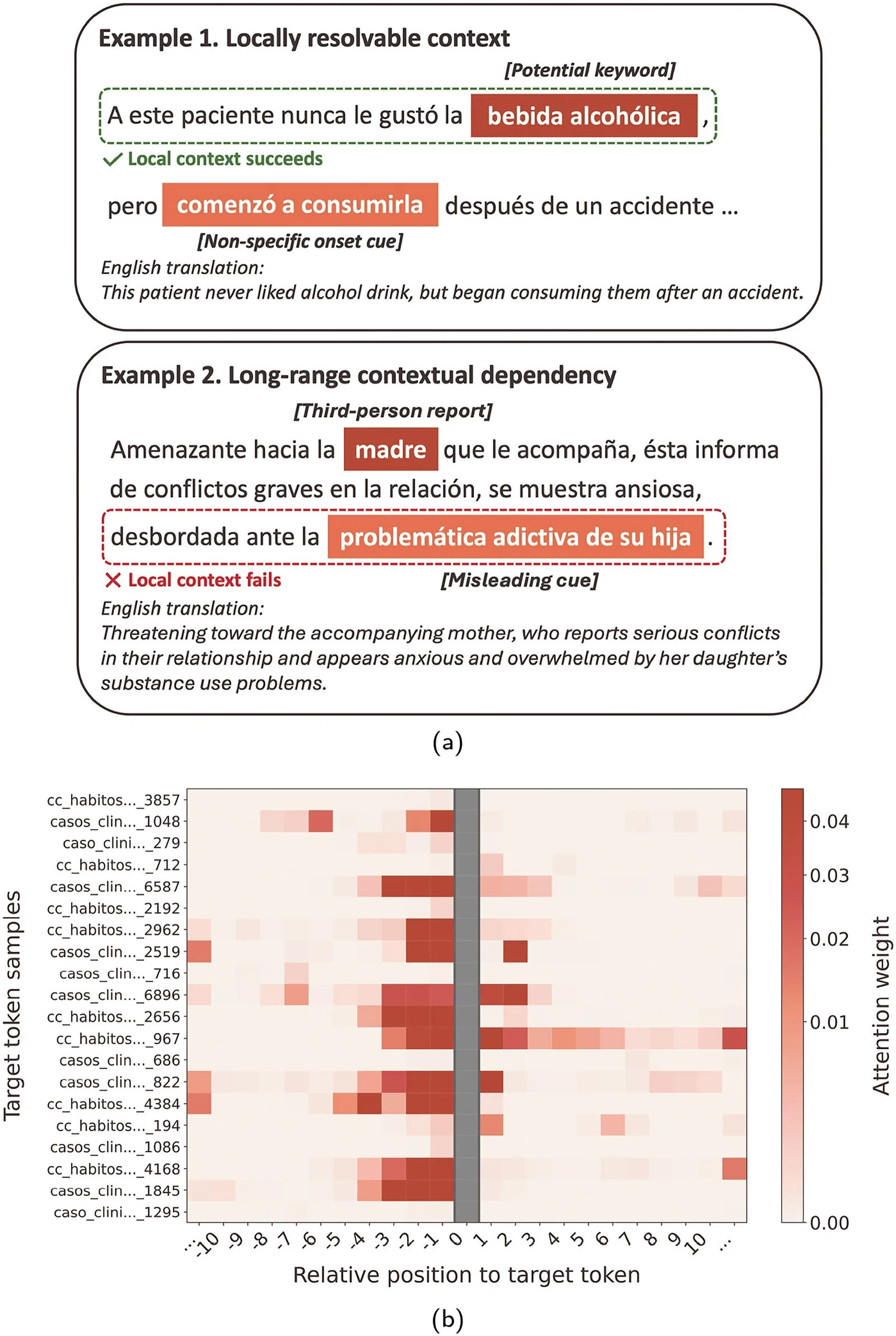
Qualitative and quantitative analysis of contextual scope in substance use NER. (a) Illustrative examples of heterogeneous contextual dependencies. (b) Aggregated token-level attention heatmap centred on the target mention (offset 0; grey band). The x-axis shows relative token offset (negative: previous context; positive: following context). The y-axis indexes sampled instances by source document ID (truncated). Colour intensity indicates attention weight (darker: higher).Please use the standard alt text.

**Structural and contextual challenges** arise because detecting a substance use mention often depends on cues beyond the entity boundary. Figure 1 presents qualitative and quantitative analyses of these challenges. Figure 1a illustrates heterogeneous contextual dependencies in clinical narratives. Clinical notes are characteristically elliptical, favouring compact expressions and clause-level constructions over fully articulated discourse; as a result, evidence for correct labelling may be concentrated in short spans. In Example 1, clause-level context suffices because the relevant modifiers are tightly coupled with the trigger, while avoiding dilution from broader sentence-level context that can suppress the decision. However, local evidence is not always sufficient. In Example 2, attributional cues indicating third-person reporting occur outside the immediate context of the target mention; restricting the model to local context may therefore lead to misinterpreting the substance use problem as referring to the patient rather than to her daughter. Figure 1b shows an aggregated attention heatmap across 20 randomly sampled test instances, measuring the distribution of model attention relative to the target mention. Attention is predominantly concentrated around the target token and nearby positions, while consistent attention signals at greater distances indicate discourse-dependent cases that rely on broader contextual cues.

These findings suggest that substance use NER cannot be reliably addressed by uniformly favouring either local or global context. Expanding context indiscriminately may introduce irrelevant information, whereas overly restrictive windows may omit essential cues. Effective extraction, therefore, requires selecting an appropriate amount of context for each instance. This motivates an *adaptive context selection strategy* in which compact context is preferred when sufficient, and longer-range cues are incorporated only when necessary. To address this challenge, we have developed an adaptive context selection (ACS) approach that prioritizes resolving immediate scope while incorporating broader discourse context when required.

In this paper, we introduce I-NOWJ, an integrated framework for substance use NER in Spanish clinical narratives. I-NOWJ is an improved version of our previously proposed NOWJ system [14], which achieved first-ranked performance in the ToxHabits shared task. Our main contributions can be summarized as follows:

- We propose a low-resource strategy that combines domain knowledge expansion and data-efficient learning through controlled data augmentation and quality-aware self-training.
- We introduce an ACS approach that dynamically selects compact or extended context depending on instance-specific requirements.

The remainder of this paper is organized as follows. The *Related work* section reviews related work. The *Materials and methods* section describes the ToxHabits corpus and the I-NOWJ framework. The *Results and discussion* section presents the experimental results and discussion. Finally, the *Conclusion* section concludes the paper.

### Related work

#### Clinical NER

Biomedical NER has achieved strong performance for common entity types such as genes, diseases, chemicals, and proteins [15, 16]. Recent progress has been driven largely by encoder-based Transformer models pretrained on domain-specific corpora [17, 18]. These models leverage annotated biomedical text during pretraining, enabling more accurate contextual encoding of specialized terminology and improving generalization across diverse biomedical benchmarks. Recent work has begun to address the extraction of substance use-related information from clinical narratives, such as annotating opioid use disorder severity from electronic health records [19]. However, this line of research remains comparatively limited relative to mainstream clinical NER tasks [19, 20]. Although recent advances in large language models (LLMs) have demonstrated strong performance in reasoning-intensive biomedical tasks, they remain less reliable for token-level sequence labelling, such as NER [21, 22]. Empirical evidence indicates that difficulties in predicting precise entity spans in clinical text, particularly for specialized or rare entity types [23, 24], constitute a substantial proportion of recognition mistakes. Motivated by these challenges, we formulate the task as a sequence labelling problem to enable precise token-level span prediction.

#### Data augmentation in low-resource NER

Data augmentation has become a widely adopted strategy to alleviate data scarcity in NLP, particularly in low-resource settings [25, 26]. A range of techniques, including distant supervision, backtranslation, generative approaches, and others, have been explored to enhance robustness and generalization in NER.

Distant supervision with silver standard data has been explored as an effective strategy for improving NER performance [16, 27, 28]. By leveraging external knowledge bases to label large collections of unlabelled documents automatically, it can substantially expand training data without manual annotation. However, this approach typically depends on the availability of comprehensive and well-curated knowledge bases, as well as access to large document repositories from which reliable silver data can be extracted [27]. Such prerequisites are often difficult to satisfy in low-resource languages such as Spanish.

Backtranslation [29] has been widely used to introduce lexical and syntactic diversity in low-resource scenarios. However, repeated translation may introduce subtle semantic shifts in the surrounding context, potentially altering the original meaning of the sentence. Such semantic drift is particularly problematic for substance use extraction, where accurate labelling depends heavily on contextual cues rather than isolated token-level signals.

More recently, LLMs have been employed to rewrite original sentences in order to enrich training data [30, 31]. Nevertheless, these approaches often focus on increasing the quantity of generated samples without explicitly preserving semantic dependencies among related entities or accounting for contextual shifts, both of which are critical for substance use NER. As a result, augmented sentences may deviate from the original discourse structure or disrupt interactions between triggers and their associated arguments. Consequently, LLM-based augmentation may inadvertently introduce noise rather than provide reliable supervision.

Lexical substitution based on word similarity has been extensively explored as a data augmentation strategy [32–34]. By replacing tokens with semantically related alternatives derived from embedding spaces or lexical resources, this approach increases surface-level lexical diversity. However, such substitutions typically optimize token-level similarity in latent spaces without explicitly modelling token-context semantic dependencies, which may distort subtle contextual cues critical for clinical NER and may introduce noisy or semantically inconsistent samples [32]. Self-training is also widely adopted in low-resource scenarios [28,35]. While this strategy can expand supervision and improve performance, its effectiveness strongly depends on the reliability of initial predictions; noisy pseudo-labels may propagate errors and amplify training noise, particularly in domain-specific settings.

For substance use NER, augmentation must preserve dependencies between triggers and their associated arguments, as well as contextual cues such as negation and temporality. This highlights the need for context-preserving and low-resource-aware augmentation strategies.

#### Knowledge integration

Domain knowledge integration has become a widely adopted strategy for domain-specific and low-resource NER, leveraging external resources such as biomedical lexicons, ontologies, and curated dictionaries to improve lexical coverage and semantic consistency. Early efforts incorporated dictionary-derived features at the token or span level, demonstrating that explicit lexicon signals can regularize neural encoders and enhance the recognition of rare or unseen entities by injecting prior domain knowledge [36]. Subsequent studies advanced this line of work by introducing auxiliary gazetteer modules into neural NER systems, allowing external knowledge to be fused with contextual representations and reinforcing entity predictions, particularly in low-resource scenarios [37]. More recent approaches reformulate lexicon integration as a structured modelling problem. Dictionary-matching graph networks, for instance, represent matched spans as nodes within graph structures and apply graph convolution to capture interactions among entity candidates, achieving greater robustness than conventional lookup-based mechanisms [38]. Recent work continues to explore deeper integration of domain-specific lexicons into pre-trained encoders to enhance boundary detection and semantic representation in medical NER [39].

Despite these advances, most lexicon-based methods remain heavily dependent on the coverage and quality of the underlying resources. While effective for formal and standardized terminology, they struggle to generalize to slang, informal expressions, and rapidly evolving variants. Alias expansion based on embedding similarity also tends to fail to capture non-compositional or brand-specific substance names. Furthermore, empirical success has predominantly been demonstrated on English-language corpora [7]. In contrast, languages such as Spanish often lack equally comprehensive and standardized biomedical lexicons, limiting the transferability and practical applicability of knowledge integration techniques in truly low-resource settings.

#### Context modelling

Most existing methods incorporate full sentences or extended surrounding contexts as inputs to neural networks, assuming that increasing contextual scope consistently improves prediction quality by exposing models to richer semantic cues and broader discourse information [20, 40]. To further extend contextual coverage, long-context Transformer architectures have been proposed to process thousands of tokens using sparse or hybrid local–global attention mechanisms [41]. While enabling document-level attention, these models often introduce substantial noise and computational overhead. In contrast, many information extraction and sequence labelling approaches rely on a fixed-size context window, which predefines token- or sentence-level spans to balance efficiency and coverage under practical memory constraints [42]. However, such static windows remain inflexible to instance-specific semantic dependencies and may fail to capture relevant evidence that falls outside predefined boundaries. Sliding-window and chunk-based strategies further partition long sequences into overlapping local segments to control memory usage [43], yet they frequently disrupt long-range contextual relationships. Only limited prior work explores dynamic context selection in a data-driven manner. For example, reinforcement learning-based approaches adaptively determine contextual scope for each instance [44]. Nevertheless, instance-specific adaptive context modelling remains largely underexplored.

## Materials and methods

### Materials

In this section, we first introduce the ToxHabits dataset, which is used as the main benchmark for training and evaluation. We then describe the construction of the domain-specific knowledge bases developed to support our approach.

#### ToxHabits dataset

The ToxHabits dataset is a specialized Spanish clinical corpus introduced during the BioCreative IX workshop to facilitate the automatic extraction of substance use information [4]. The corpus employs an event-based annotation scheme in which clinical experts have manually identified substance use mentions, designated as triggers, and their corresponding contextual attributes, referred to as arguments. These entities are categorized as follows:

- Triggers mentions are classified into four categories: Alcohol, Tobacco, Cannabis, and Drug—representing all other psychoactive substances.
- Argument mentions are classified into six categories:
  ○ Type: Specifies the particular substance used, e.g. *cocaína* (‘cocaine’), *heroína* (‘heroin’).
  ○ Method: Describes the route of administration, e.g. *fumada* (‘smoked’) and *inyectada* (‘injected’).
  ○ Amount: Indicates the quantity consumed, e.g. *dos cervezas* (‘two beers’) and *un paquete al día* (‘a pack per day’).
  ○ Frequency: Details the regularity of use, e.g. *cada fin de semana* (‘every weekend’) and *a diario* (‘daily’).
  ○ Duration: Defines the time span of substance use, e.g. *durante cinco años* (‘for five years’).
  ○ History: Records the status or point in time when consumption ceased, e.g. *dejado en 2007* (‘quit in 2007’).

The ToxHabits corpus comprises 1499 clinical case reports from diverse medical specialties and reflects real-world clinical narratives documenting substance use mentions. It is partitioned into a training set of 1199 documents and a test set of 300 documents. The detailed corpus statistics are reported in Table 1.

**Table 1:** Descriptive statistics of the ToxHabits dataset.

Metric	Train	Test
Number of documents	1199	300
Average document length (words)	516	498
Average sentences per document	26.85	24.57
Total triggers	7592	1838
Total arguments	9528	2388
Mean triggers per document	6.33	6.13
Mean arguments per document	7.95	7.96

#### Domain knowledge bases

This section presents the construction of domain knowledge bases that will be integrated into the knowledge-infused representation phase of our proposed model (the *Knowledge-infused representation phase* section). Since triggers and arguments are treated as distinct entity types, we construct two separate dictionaries: a trigger dictionary covering substance use mentions and an argument dictionary covering usage characteristics.

**Trigger dictionary construction.** We leverage domain-specific external resources to construct a comprehensive trigger lexicon for substance use NER in Spanish. The lexicon aggregates various surface forms of pharmacological and psychoactive substances, including canonical names, synonyms, and lexical variants commonly observed in free-text clinical narratives. It is constructed through the systematic integration of multiple complementary domain-specific resources.

- A standardized foundation is established by extracting drug name strings from the DrugBank vocabulary dataset (DrugBank open data, version 5.1.13, downloaded in July 2025) [45].
- Unique drug name mentions from the DDIExtraction 2013 corpus [46] are also collected to capture mention forms attested in biomedical literature.
- Additional coverage for non-canonical spellings and informal references is provided through the incorporation of the RedMed lexicon [47], which maps consumer health and social media expressions to standardized biomedical identifiers.
- To improve recall for long-tail surface forms that are underrepresented in conventional terminologies, open-access web-derived substance use trigger lists (hereafter referred to as “Web Data”) are incorporated. Prominent subsets within these web-derived lists include FDA drug names (FDA drug name: https://www.fda.gov/drugs/drug-approvals-and-databases/drugsfda-data-files, accessed 15 July 2025), the Kaggle Illicit Drugs dataset (Kaggle Illicit Drugs: https://www.kaggle.com/datasets/willianoliveiragibin/illicit-drugs, version 1, accessed 15 July 2025), and two Wikipedia lists of slang names for cannabis and alcoholic drinks (Wikipedia list of slang names for cannabis: https://en.wikipedia.org/wiki/List_of_slang_names_for_cannabis, accessed 15 July 2025) (Wikipedia list of alcoholic drinks: https://en.wikipedia.org/wiki/List_of_alcoholic_drinks, accessed 15 July 2025).

Curated trigger strings harvested from external resources are exclusively in English. We first apply Unicode normalization, lowercasing, and de-duplication to reduce redundant surface forms and lexical noise. We then batch the normalized English trigger terms and prompt Gemini 2.0 Flash (Gemini 2.0 Flash model documentation: https://docs.cloud.google.com/vertex-ai/generative-ai/docs/models/gemini/2-0-flash, accessed 26 February 2026) to translate them into Spanish and to generate Spanish aliases for each term, capturing common surface-form variants in clinical narratives. The LLM outputs are subsequently re-normalized, lowercased, and de-duplicated, and the resulting Spanish inventory is merged with the trigger surface forms observed in the annotated training split to obtain a unified trigger dictionary.

In total, we construct a trigger dictionary comprising 18 856 entries; detailed analyses and statistics are provided in the *Knowledge base analysis* section.

**Argument dictionary construction**. Argument entities exhibit heterogeneous textual expressions and lack standardized external resources comparable to pharmacological lexicons. We therefore derive the argument dictionary from the annotated ToxHabits training split and expand it with a prompt-constrained LLM, Gemini 2.0 Flash (Gemini 2.0 Flash model documentation: https://docs.cloud.google.com/vertex-ai/generative-ai/docs/models/gemini/2-0-flash, accessed 26 February 2026). Specifically, for each argument mention in the training set, we provide the model with the mention and one training sentence in which it occurs and instruct it to generate a small set of plausible Spanish variants that could express the same argument in similar contexts. Generated variants inherit the category of their seed mention, preventing the expansion from introducing new label types. To reduce sparsity induced by numeric expressions, we require the model to abstract numbers and years as <NUM> and <YEAR> during generation, and we apply the same deterministic abstraction to both seeds and generated variants. We then perform Unicode normalization, lowercasing, and deduplication before merging the resulting candidates with the argument surface forms extracted from the annotated training split to form the final argument dictionary. In total, we construct an argument dictionary comprising 15 297 entries; detailed analyses and statistics are provided in the *Knowledge base analysis* section.

In total, we construct an argument dictionary comprising 15 297 entries; detailed analyses and statistics are provided in the *Knowledge base analysis* section.

### Proposed model

Both trigger and argument extraction are formulated as sequence labelling problems under the BIO (Begin–Inside–Outside) tagging scheme [48], in which labels prefixed with B- and I- indicate the beginning and inside positions of a span corresponding to a trigger or argument category, respectively, while O denotes tokens outside any labelled span. Given an input clinical sequence $S = \lbrace x_1, x_2, \dots , x_n\rbrace$ of *n* tokens, the task is divided into two primary subtasks and addressed under a multi-task learning framework with shared contextual representations and a joint loss objective.

1. Trigger extraction: For each token $x_i$, the model assigns a label $y^{trig}_i \in \mathcal {Y}_{trig}$, where $\mathcal {Y}_{trig} = \lbrace B\mathrm{-}t, I\mathrm{-}t \mid t \in \mathcal {T}\rbrace \cup \lbrace O\rbrace$. Here, $\mathcal {T} = \lbrace \mathtt {Alcohol, Tobacco, Cannabis, Drug}\rbrace$ represents the set of trigger categories.
2. Argument extraction: For each token $x_i$, model assigns a label $y^{arg}_i \in \mathcal {Y}_{arg}$, where $\mathcal {Y}_{arg} = \lbrace B\mathrm{-}a, I\mathrm{-}a \mid a \in \mathcal {A}\rbrace \cup \lbrace O\rbrace$. The set $\mathcal {A} = \lbrace \mathtt {Type, Method, Amount, Frequency, Duration}\rbrace$ History represents the set of argument categories.

**Figure 2:**
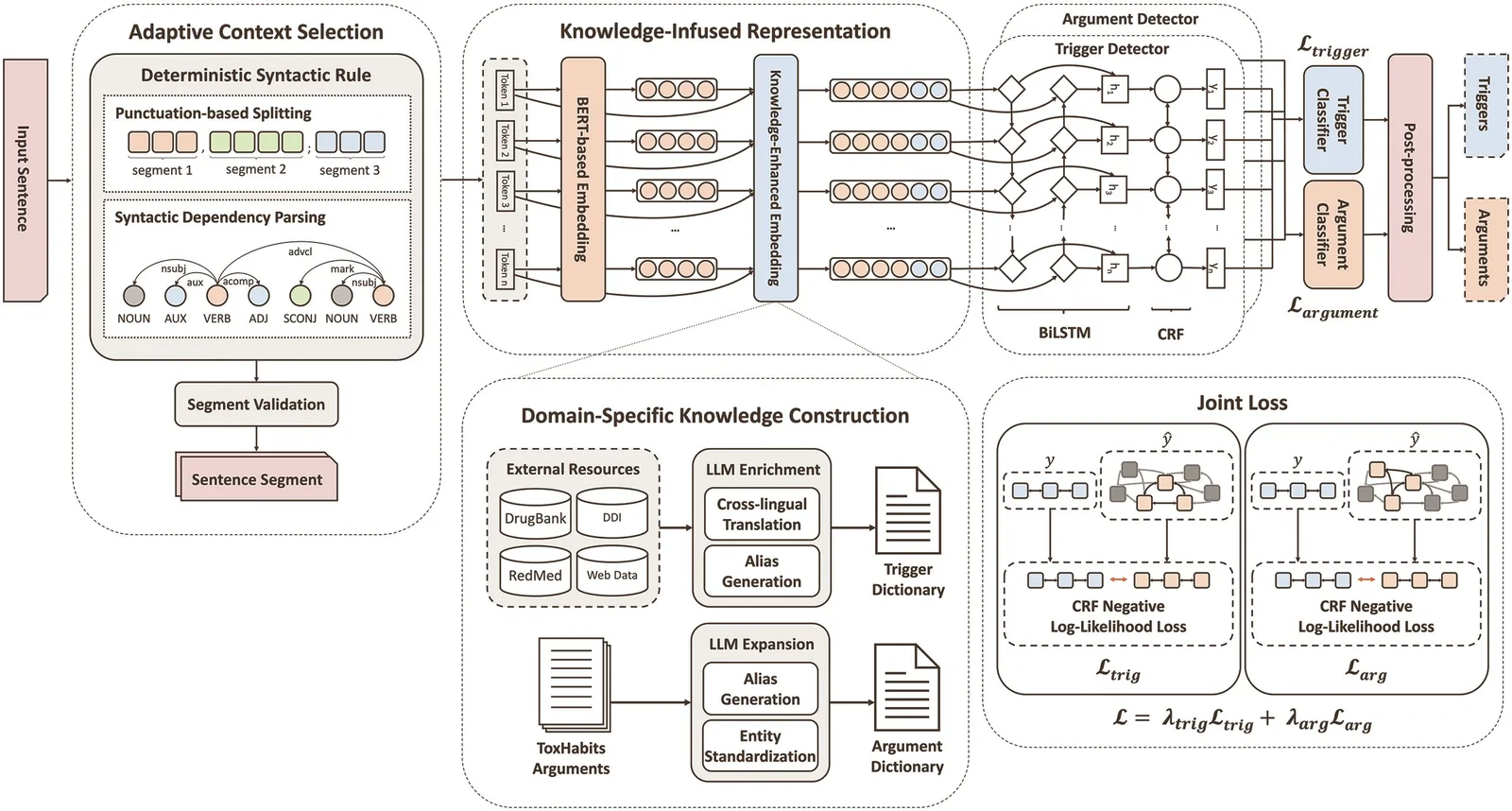
Overview of the I-NOWJ architecture. The framework consists of three main phases: adaptive context selection, knowledge-infused representation, and trigger–argument detection.

Figure 2 illustrates the I-NOWJ framework, which comprises three main phases: ACS, knowledge-infused representation, and trigger–argument detection. Each phase is described in the following subsections.

#### Adaptive context selection phase

The ACS phase addresses a limitation in substance use NER: the assumption that a single, fixed context granularity is universally optimal. Indiscriminate context expansion risks introducing semantically irrelevant noise, whereas overly restrictive windowing may discard critical cross-clausal dependencies required for accurate entity recognition.

To navigate this trade-off, we propose a deterministic, syntax-driven segmentation strategy. Rather than relying on predefined context windows, the algorithm dynamically partitions each sentence into syntactically complete clauses only when dependency parsing confirms that every resulting segment contains a verbal predicate with an associated subject relation. Otherwise, the original sentence is preserved as an indivisible context unit. The procedure is formalized in Algorithm 1.

**Algorithm 1:**
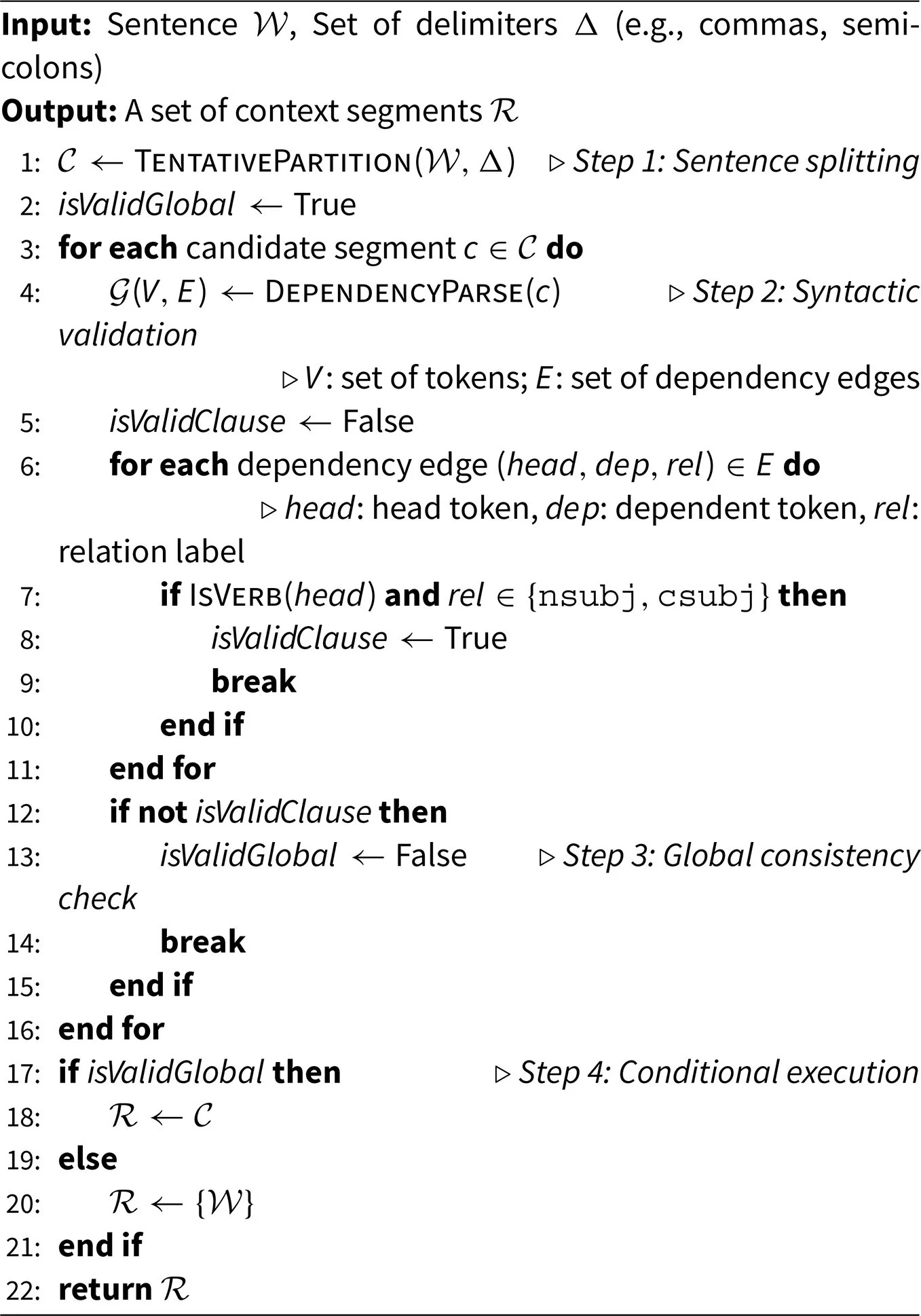
Adaptive Context Selection via Syntactic Constraints

**Figure 3:**
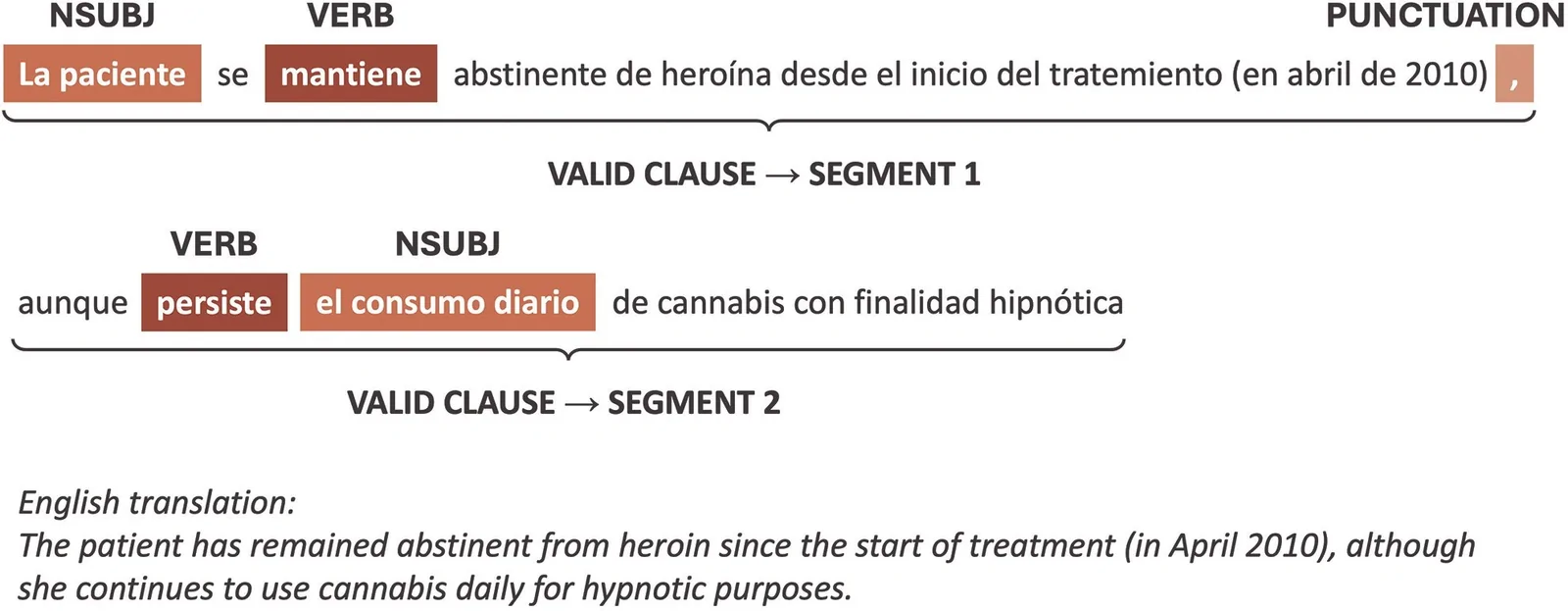
An example of our adaptive context selection scheme.

Figure 3 illustrates how the ACS strategy operates on a complex clinical narrative. In this example, a sentence containing multiple interleaved clinical observations is conditionally decomposed into two semantically complete fragments that satisfy the predefined syntactic validity criteria.

#### Knowledge-infused representation phase

In this phase, token representations are enriched with external domain knowledge to improve robustness to lexical variability in Spanish clinical narratives. Firstly, for semantic representations, we feed the input sentence *S* into a pre-trained Spanish BERT encoder [49]. For each token $x_i$, the encoder produces a contextualized embedding $h_i^{B} \in \mathbb {R}^{d_B}$ that captures token-level semantics within its surrounding context, where $d_B$ is the hidden dimension of the Spanish BERT encoder.

To infuse explicit domain knowledge into token representations, we perform span matching over the trigger and argument dictionaries (the *Domain knowledge bases* section) to obtain category-level lexical indicators aligned with the token sequence. For each token $x_i$, we construct a binary sparse feature vector $k_i$ indicating whether the token falls inside a matched trigger or argument span of each category:


(1) \begin{eqnarray*}
k_i \in \lbrace 0,1\rbrace ^{|\mathcal {T}| + |\mathcal {A}|}
\end{eqnarray*}

where $\mathcal {T}$ and $\mathcal {A}$ denote the trigger and argument category sets, respectively; therefore, $|\mathcal {T}|+|\mathcal {A}|$ is the number of category-level indicator dimensions in $k_i$. Each dimension corresponds to one trigger or argument category and is set to 1 when $x_i$ belongs to a dictionary-matched span of that category, and 0 otherwise. Rather than directly concatenating these sparse features, we project $k_i$ into a dense representation space using a two-layer MLP. This transformation enables the model to learn smooth interactions between lexical categories and mitigates the rigidity of hard dictionary constraints. The resulting vector, denoted as $h_i^{k}$, serves as the knowledge-based embedding for token $x_i$:


(2) \begin{eqnarray*}
\ h_i^{k} = W_2 \cdot \mathrm{ReLU}(W_1 k_i + b_1) + b_2.
\end{eqnarray*}

Here, $h_i^{k} \in \mathbb {R}^{d_k}$. $W_1 \in \mathbb {R}^{d \times (|\mathcal {T}|+|\mathcal {A}|)}$ and $W_2 \in \mathbb {R}^{d_k \times d}$ are trainable weight matrices, while $b_1 \in \mathbb {R}^{d}$ and $b_2 \in \mathbb {R}^{d_k}$ are trainable bias vectors. $d_k$ denotes the dimension of the knowledge vector, while *d* represents the hidden size of the first linear layer in the MLP.

The knowledge-based embedding is then concatenated with the semantic representation. Formally, the final knowledge-infused embedding of the token at position *i* is:


(3) \begin{eqnarray*}
h_i = h_i^{B} \oplus h_i^{k}
\end{eqnarray*}

where $h_i \in \mathbb {R}^{d_B + d_k}$ and *⊕* denotes vector concatenation.

These representations are then used as the input to the subsequent sequence modelling phase.

#### Trigger–argument detectors

Building upon the knowledge-infused representations, this phase models long-range contextual dependencies and enforces structural consistency in sequence labelling. The sequence of token representations $\lbrace h_1, \dots , h_n\rbrace$ is passed through a bidirectional long short-term memory (BiLSTM) network [50], which captures both preceding and succeeding contextual information at each position:


(4) \begin{eqnarray*}
z_i = \phi _{\mathrm{fwd}}(h_i, z_{i-1}) \oplus \phi _{\mathrm{bwd}}(h_i, z_{i+1})
\end{eqnarray*}

where $\phi _{\mathrm{fwd}}$ and $\phi _{\mathrm{bwd}}$ denote the forward and backward LSTM functions, respectively, and $z_i \in \mathbb {R}^{2d_l}$ is the contextualized representation at position *i*. Here, $d_l$ denotes the hidden dimension of each unidirectional LSTM.

To enforce valid label transitions in span-based extraction, we employ two task-specific conditional random field (CRF) layers [51] over the BiLSTM outputs [52]: one for trigger extraction and one for argument extraction, each operating over the full label sequence. Both CRF layers use the same formulation but have independent parameters. For each task, given the contextualized representations $\lbrace z_1, \dots , z_n\rbrace$ and a candidate label sequence $y = (y_1, \dots , y_n)$, the CRF defines the conditional probability:


(5) \begin{eqnarray*}
P(y \mid z_{1:n}) = \frac{1}{Z(z_{1:n})} \exp \left(\sum _{i=1}^{n} \psi _e(y_i, z_i) + \sum _{i=2}^{n} \psi _t(y_{i-1}, y_i)\right)
\end{eqnarray*}

where $\psi _e(y_i, z_i)$ denotes the task-specific emission score derived from the contextualized representation $z_i$, $\psi _t(y_{i-1}, y_i)$ represents the task-specific transition score capturing dependencies between adjacent labels, and $Z(z_{1:n})$ is the partition function that normalizes over all possible label sequences. The transition scores are particularly critical for enforcing valid BIO constraints, such as preventing an I- tag from following an incompatible B- tag. By modelling label dependencies globally, the CRF layer improves sequence-level coherence compared to independent token classification.

The model is trained by maximizing the conditional log likelihood of the gold label sequence. During inference, Viterbi decoding [53] is applied to recover the globally optimal label sequence, and the predicted BIO tags are subsequently converted into explicit entity spans through deterministic boundary aggregation.

#### Joint learning and low-resource strategy

This section presents the learning strategy designed to improve robustness and generalization. We introduce a multi-task learning framework with joint loss, a context-aware data augmentation approach coupled with self-training, and an ensemble mechanism for prediction refinement.

##### Multi-task learning scheme with joint loss

Triggers and their corresponding arguments are semantically interdependent and frequently co-occur within shared contextual spans. We therefore formulate both extraction tasks under a unified multi-task learning framework, enabling the model to jointly capture their hierarchical and semantic dependencies while producing trigger and argument labels for each token simultaneously.

Let $y^{\mathrm{trig}} = (y_1^{\mathrm{trig}}, \dots , y_n^{\mathrm{trig}})$ and $y^{\mathrm{arg}} = (y_1^{\mathrm{arg}}, \dots , y_n^{\mathrm{arg}})$ denote the gold label sequences for trigger and argument extraction, respectively. The training objective for each task is defined as the negative conditional log likelihood of its corresponding CRF:


(6) \begin{eqnarray*}
\mathcal {L}_{\mathrm{trig}} = -\log P(y^{\mathrm{trig}} \mid z_{1:n}), \quad \mathcal {L}_{\mathrm{arg}} = -\log P(y^{\mathrm{arg}} \mid z_{1:n})
\end{eqnarray*}

where $z_{1:n}$ denotes the contextualized representations produced by the BiLSTM. The overall training objective is a weighted combination of both losses:


(7) \begin{eqnarray*}
\mathcal {L} = \lambda _{\mathrm{trig}} \, \mathcal {L}_{\mathrm{trig}} + \lambda _{\mathrm{arg}} \, \mathcal {L}_{\mathrm{arg}}
\end{eqnarray*}

where $\lambda _{\mathrm{trig}}$ and $\lambda _{\mathrm{arg}}$ are non-negative hyperparameters that control the relative contribution of each subtask. This weighting scheme provides flexibility to balance the learning dynamics between trigger and argument extraction, accommodating potential disparities in task difficulty or label distribution.

##### Data augmentation and self-training

**Figure 4:**
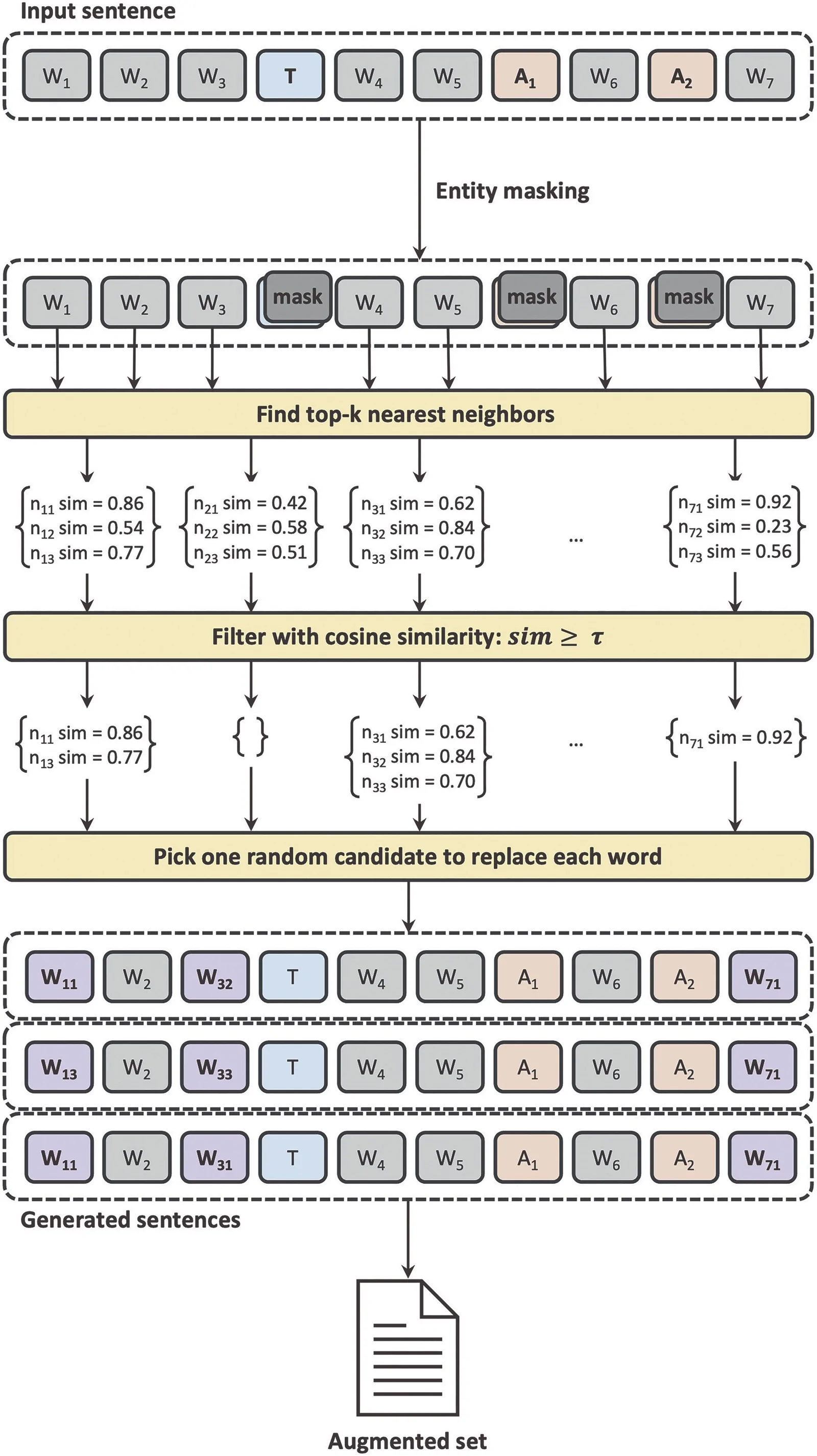
Overview of the proposed context-aware data augmentation pipeline. Sentences containing labelled entities are selected and tokenized. ‘T’ and ‘A’ denote trigger and argument entities, respectively, while ‘W’ represents non-entity tokens.

To alleviate data sparsity and the complexity of trigger and argument extraction, we propose a context-aware data augmentation strategy (Fig. 4). Training samples containing labelled entities are tokenized at the word level, and entity spans are masked to prevent modification and preserve annotation consistency. For each non-entity token, we retrieve its top-*m* nearest neighbours in a pre-trained Spanish fastText [54] embedding space. Candidates are filtered using a predefined cosine similarity threshold *τ*, where *τ* is the minimum similarity required for replacement eligibility. A replacement token is then randomly sampled from the candidates that exceed this threshold. By applying these substitutions independently to multiple non-entity tokens, we generate diverse augmented instances while preserving the original entity annotations.

To mitigate noise introduced during augmentation, we employ a self-training strategy. We first train the model on the original labelled dataset and then use it to predict labels for the augmented corpus. For each augmented sentence, we retain only instances whose predicted labels remain fully consistent with the entity annotations transferred from the corresponding source sentence. The filtered samples are merged with the original labelled dataset to form an expanded training set. Subsequently, a completely new model is initialized and trained on the updated dataset to avoid bias accumulation from the previous model parameters. This iterative consistency filtering suppresses low-quality synthetic instances and alleviates semantic drift introduced during contextual substitution. After the refinement round, the final model is trained on the resulting high-quality dataset to obtain more robust and generalizable representations.

##### Ensemble mechanism

To reduce performance variance caused by random initialization in this low-resource clinical NER setting, we adopt a seed-based ensemble strategy. Specifically, *K* instances of the I-NOWJ architecture are trained using identical hyperparameters and training data, differing only in their random initialization seeds, where *K* denotes the ensemble size. Although the models share the same design, each run converges to slightly different decision boundaries, particularly for ambiguous trigger–argument spans and uncertain entity boundaries. During inference, predictions are aggregated via majority voting at the entity level. An entity is included in the final output only if it is predicted by at least $K_0$ models, where $K_0$ is the entity-level voting threshold; otherwise, it is discarded.

## Results

### Experimental settings

The BioCreative IX ToxHabits shared task defines micro-averaged precision, recall, and F1 score as the official evaluation metrics for both subtasks, ToxNER and ToxUse. A prediction is considered correct only if both its span and type exactly match the gold annotation [4]. In some experiments, we additionally report an overlap-based metric to provide a more fine-grained analysis. The official training and testing splits used in our experiments are described in the *ToxHabits dataset* section.

Our multi-task learning framework employs a pretrained contextual encoder to generate token-level representations for both subtasks. We adopt the BERT-base model *bert-base-spanish-wwm-cased* [49] as the backbone and fine-tune it end-to-end with all downstream components. We use the spaCy pipeline (version 3.7.2: https://zenodo.org/records/10009823) for word-level tokenization and dependency parsing. The full set of training hyperparameters used in all experiments is reported in Table 2.

**Table 2:** Training setup and hyperparameter configuration used in all experiments.

Component	Value	Hyperparameter	Value
*Dictionary/representation dimensions*		*BiLSTM*	
BERT hidden size ($d_B$)	768	Hidden size ($d_l$)	256
Knowledge MLP hidden size (*d*)	32	Layers	2
Knowledge embedding dim ($d_k$)	128	Dropout	0.2
*Data augmentation and self-training*		*Learning rates*	
Top-*m* neighbours (*m*)	5	BERT	$2 \times 10^{-5}$
Cosine threshold (*τ*)	0.7	BiLSTM	$5 \times 10^{-4}$
Self-training iterations	1	Classifier	$1 \times 10^{-3}$
*Ensembling*		*Optimization*	
Ensemble size (*K*)	5	Weight decay	0.01
Voting threshold ($K_0$)	3	Warm-up steps	*10%*
*Loss weighting*		*Training schedule*	
Trigger weight ($\lambda _{\mathrm{trig}}$)	1.0	Batch size	16
Argument weight ($\lambda _{\mathrm{arg}}$)	1.0	Training epochs	7

### Results and comparative analysis

This section presents a comprehensive empirical evaluation of the proposed approach. We report overall performance and comparisons with competing systems, followed by ablation studies and component-wise analyses to assess the contribution of key modelling and training strategies. Finally, we provide error analysis and discuss limitations.

#### Overall performance

The BioCreative IX ToxHabits participating teams predominantly adopted two modelling directions: supervised fine-tuning of pre-trained language models and generative approaches using LLMs. Systems prioritizing supervised learning, such as those by ICB-UMA [55], GooseSeek, and Orekhovichi [56], leveraged domain-specific encoders (e.g. biomedical RoBERTa [57], BETO) often coupled with CRF layers or span-marker classification. Conversely, teams like SINAI [58] and FMI-SU [59] explored hybrid or few-shot prompting techniques using generative models (e.g. GPT-4, DeepSeek). The comparative results for the official shared task evaluation are presented in Table 3. Our official competition submission, NOWJ, achieved the highest performance in both subtasks, recording an F1 score of *94%* for trigger extraction and *91%* for argument extraction.

**Table 3:** Official evaluation results for Subtask 1 (ToxNER—trigger) and Subtask 2 (ToxUse—argument).^a^

		Trigger (ToxNER)			Argument (ToxUse)		
Team	Technique	P	R	F1	P	R	F1
NOWJ (official run)	BETO + CRF ensemble	94	94	94	91	90	91
SINAI	EuroBERT + LLM few-shot	89	89	89	78	79	78
ICB-UMA	Span-based RoBERTa ensemble	83	85	84	73	77	75
GooseSeek	Weighted RoBERTa fine-tuning	87	72	79	75	66	70
Orekhovichi	BERT-CRF + data augmentation	83	65	73	47	60	53
FMI-SU	GPT-4.1 few-shot prompting	59	72	65	–	–	–
**I-NOWJ (proposed)**	NOWJ + adaptive context selection + knowledge-guided learning	**94.72**	**95.14**	**94.93**	**92.84**	**89.80**	**91.30**
*I-NOWJ*	*Overlap-based matching* ^b^	*95.71*	*96.13*	*95.92*	*96.76*	*93.37*	*95.03*

a All scores are reported as percentage (%). The official scores are provided in decimal format and rounded to two decimal places, thereby truncating finer-grained variations. For clearer comparison, we present all results as percentages to two decimal places.

b A predicted span is considered correct if it overlaps with the gold span and the predicted type matches the gold label.

Building upon this strong baseline, we developed I-NOWJ to address the residual errors observed in the competition phase. The I-NOWJ architecture consistently outperforms the original NOWJ system, yielding an improvement of ∼*1%* across Precision, Recall, and F1-score metrics for subtask ToxNER (trigger extraction) and *2%* across Precision for subtask ToxUse (argument extraction). This consistent performance motivates explicit modelling of adaptive context and the incorporation of external knowledge as an auxiliary signal to further improve trigger and argument extraction in complex clinical texts.

Beyond the official exact-match evaluation, we additionally assess our model under a relaxed overlap-based criterion, shown in the last row of Table 3. This metric captures the model’s ability to localize relevant spans even when boundaries are not perfectly aligned. As expected, overlap-based evaluation yields higher scores for both subtasks. For trigger extraction, the micro-averaged F1 score increases by ∼1–95.92%. Argument extraction benefits more substantially, with F1 rising from 91.30–95.03%, indicating that precise boundary delineation is more challenging for arguments than for triggers.

#### Ablation studies

**Figure 5:**
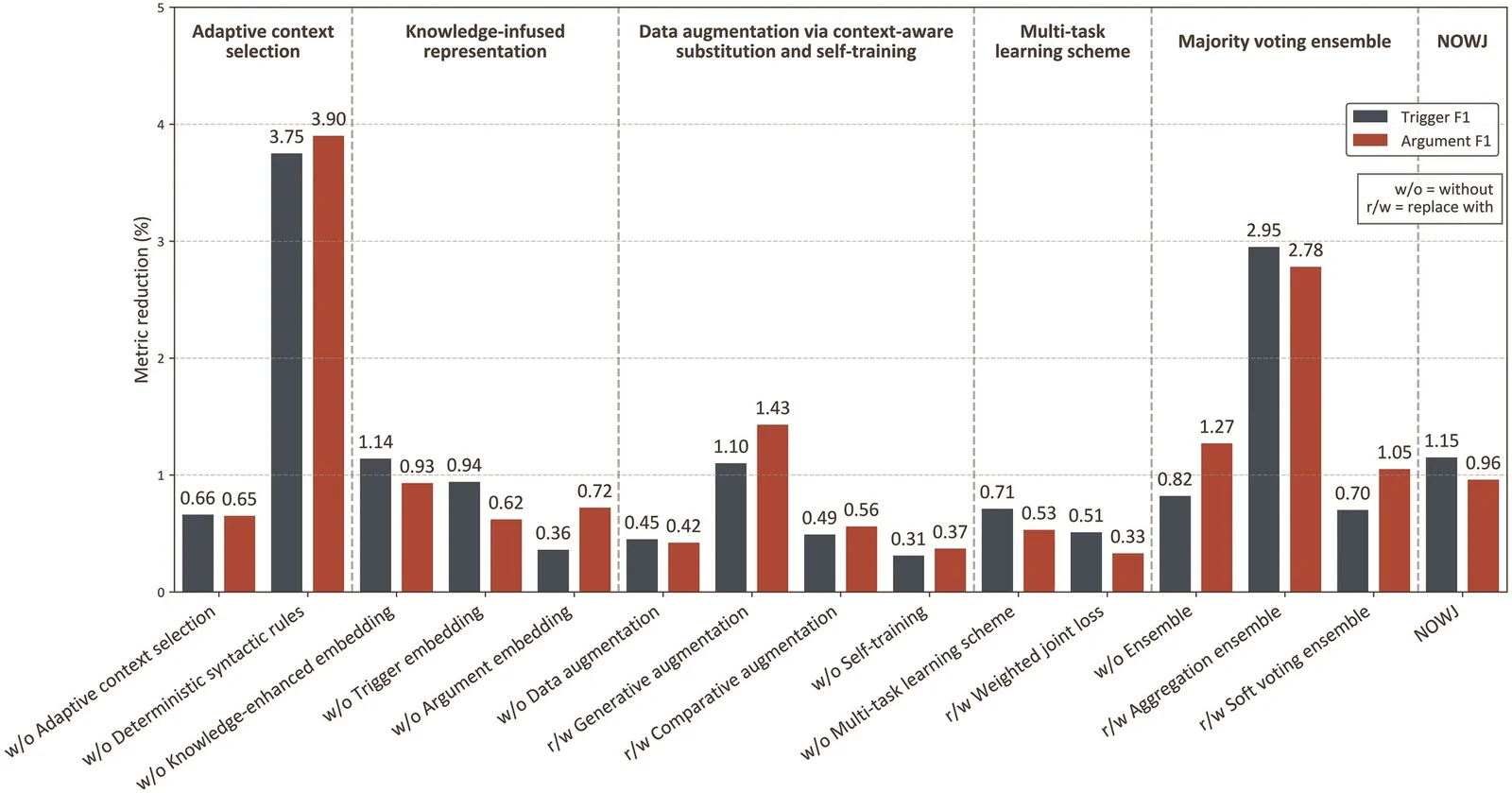
Ablation analysis quantifying the performance degradation (in F1 score) when removing (*w/o*) or replacing (*r/w*) key components of the I-NOWJ framework. All values are reported in percentage points (%).

To evaluate the impact of each phase in our framework, we perform an ablation study, reporting absolute decreases in trigger and argument F1, expressed in percentage points, when specific components are removed (*w/o*) or replaced (*r/w*). Figure 5 presents the performance changes under different ablation settings, highlighting that all components contribute to the model’s performance to varying degrees across subtasks.

##### Adaptive context selection phase

The ACS phase acts as a key context-regulation mechanism in I-NOWJ, guiding the model towards semantically relevant spans. Ablation results show that removing this component (*w/o ACS*) reduces trigger and argument F1 by 0.66 and 0.65 percentage points, respectively, indicating difficulty in identifying the appropriate contextual scope. The nearly identical degradation across the two subtasks suggests that the ACS mechanism benefits trigger and argument extraction similarly by providing a more appropriate context length. A much larger drop is observed when deterministic syntactic rules are removed while retaining punctuation-based segmentation (*without deterministic syntactic rules*), with performance declining by *3.75%* and *3.90%* F1—the largest degradation in our study. This highlights the importance of syntactic constraints in preserving coherent clause-level context for accurate trigger and argument extraction.

##### Knowledge-infused representation phase

Removing this module (*w/o knowledge-enhanced embedding*) entirely causes F1 decreases of 1.14 percentage points for trigger extraction and 0.93 percentage points for argument extraction. A more detailed view shows distinct roles for the two embeddings: removing the trigger embedding leads to a degradation of *0.94%* and *0.62%*, while removing the argument embedding results in a drop of *0.36%* and *0.72%* for trigger and argument, respectively. These asymmetric degradations suggest that each embedding primarily supports its corresponding subtask, and that retaining both provides the most robust overall performance.

##### Data augmentation and self-training

The ablation results show that the effectiveness of our augmentation strategy lies in how the context is modified rather than in simply increasing the number of training samples. Removing the entire data augmentation strategy leads to a decrease of 0.45 percentage points in trigger F1 and 0.42 percentage points in argument F1. A larger drop is observed when our context-aware substitution is replaced with generative augmentation. Although generative rewriting enhances surface fluency, it may inadvertently smooth or reinterpret these domain-specific cues, leading to subtle semantic drift. Similarly, substituting our approach with comparative augmentation results in performance declines of *0.49%* in trigger F1 and *0.56%* in argument F1. Additionally, removing the self-training phase further degrades performance, showing that augmentation must be accompanied by a quality filtering mechanism.

##### Multi-task learning scheme

Using two independent single-task models leads to consistent performance drops of *0.71%* and *0.53%* F1 for trigger and argument extraction, respectively. In substance use narratives, arguments are typically semantically linked to the corresponding triggers. Joint optimization enables the shared encoder to better capture this dependency pattern. Moreover, replacing the weighted joint loss with an unbalanced formulation further reduces performance by *0.51%* for trigger F1 and *0.33%* for argument F1, underscoring the importance of properly balancing task contributions during training.

##### Ensemble mechanism

Removing the ensemble mechanism results in marked performance declines, with trigger F1 decreasing by 0.82 percentage points and argument F1 by 1.27 percentage points. Replacing the majority voting ensemble with an inclusive aggregation strategy that retains all extracted entities leads to substantial performance degradation, reducing trigger F1 by 2.95 percentage points and argument F1 by 2.78 percentage points. Similarly, the soft voting scheme, which averages token-level logits, decreases trigger F1 by 0.70 percentage points and argument F1 by 1.05 percentage points.

#### Adaptive context selection strategy analysis

##### Context selection effects

**Figure 6:**
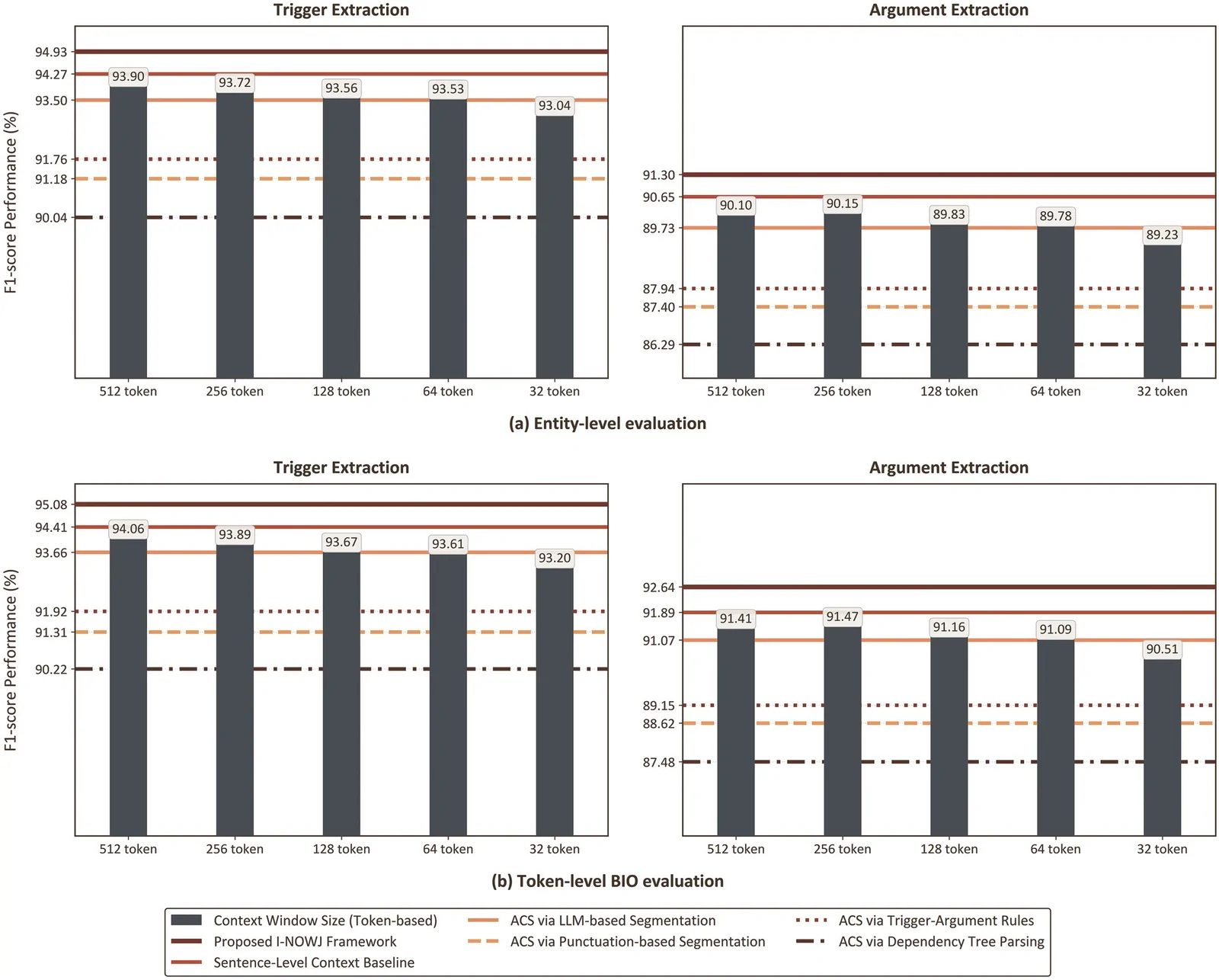
Ablation studies on context window sizes and context selection strategies. ACS denotes adaptive context selection. Panel (a) reports entity-level exact-match F1, and panel (b) reports token-level BIO F1. The bar charts show fixed context-window results, while the line plots show the corresponding F1 scores when the input contexts are decomposed into smaller segments. All scores are reported as percentage (%).

Our studies demonstrate that structurally guided ACS outperforms fixed token-based windows for substance use extraction. In addition to entity-level exact-match F1, we report token-level BIO F1 to provide a finer-grained view of label stability and boundary behaviour before span aggregation. As shown in Fig. 6a, increasing the fixed token window from 32 to 512 tokens increases the context budget 16-fold, but produces only modest gains in F1 score. Although trigger F1 increases gradually across window sizes, the marginal benefit diminishes at larger windows, with the 256-to-512 token expansion improving entity-level trigger F1 by only 0.18 percentage points. For argument extraction, the same expansion instead reduces entity-level F1. The token-level BIO results in Fig. 6b exhibit the same general pattern, indicating that the limited benefit of larger fixed windows is already observable before entity-span aggregation. Taken together, these results suggest that simply expanding the fixed context window does not consistently improve extraction performance. Notably, although the sentence-level baseline has a mean sample length of only 32.01 tokens, comparable to the smallest fixed-window setting, it still achieves higher F1 scores than all fixed-window configurations. This suggests that fixed-window limitations may arise more from arbitrary boundary placement than from insufficient context length, indicating that context quality is more important than context quantity.

To further examine this phenomenon, we evaluate alternative structural segmentation strategies. LLM-based segmentation yields semantically coherent spans, achieving *93.50%* entity-level F1 for trigger and *89.73%* entity-level F1 for argument extraction, but its boundaries rely on general semantic coherence and may vary across writing styles, leading to instability. Punctuation-based segmentation reduces noise, yet often splits trigger–argument pairs across clauses, weakening relational signals. The trigger–argument rule extension merges separated spans but may introduce irrelevant context when multiple events co-occur. Dependency parsing generates linguistically meaningful clauses but tends to produce overly fine-grained segments, excluding essential modifiers or temporal expressions. The mean segment lengths are 12.11, 12.14, 14.74, and 14.90 tokens for punctuation-based, trigger–argument rule, dependency tree parsing, and LLM-based segmentation, respectively, although their maximum lengths remain high, ranging from 150 to 167 tokens. In contrast, I-NOWJ yields moderately compact contexts, with a mean length of 19.05 tokens and a lower maximum length of 79 tokens. Together with its higher F1, these descriptive statistics are consistent with a better balance between retaining sufficient context and limiting both excessive fragmentation and unusually long segments. Overall, none of the alternative segmentation strategies surpasses the sentence-level baseline, and several underperform fixed-window configurations. For example, punctuation-based segmentation achieves *91.18%* and *87.40%* entity-level F1, while dependency parsing declines further to *90.04%* and *86.29%* entity-level F1. These findings indicate that structural segmentation alone is insufficient to ensure improvements over fixed-window settings and that rigid splitting can even degrade extraction performance. The token-level BIO results show the same general pattern, with I-NOWJ achieving the strongest performance at both the token and entity levels. Taken together, these results support adaptive, task-relevant context selection over rigid segmentation.

##### Parser sensitivity and runtime

Because ACS uses dependency parsing to validate candidate clause splits, parser errors could affect context selection and downstream entity predictions. As an external reference point, the official model card for the spaCy Spanish pipeline (results for es_core_news_sm, version 3.8.0: https://github.com/explosion/spacy-models/releases/tag/es_core_news_sm-3.8.0) reports an unlabelled attachment score (UAS) of *90.20%* and a labelled attachment score (LAS) of *86.58%* on its reference evaluation data. UAS measures whether each token is attached to the correct syntactic head, whereas LAS additionally requires the dependency relation label to be correct. Because ToxHabits does not provide gold standard dependency annotations, we cannot directly measure spaCy parsing accuracy or parser-to-NER error propagation on this corpus. We therefore assess downstream sensitivity by replacing the default spaCy backend with Stanza [60] and UDPipe [61]. As shown in Table 4, trigger F1 varies by 0.12 percentage points across the tested parsers, while argument F1 varies by 0.55 percentage points. These narrow ranges suggest that downstream extraction performance is not highly sensitive to the parser backend in this evaluation, although they do not imply that the parsers have comparable intrinsic parsing accuracy. Among the tested backends, spaCy yields the highest observed F1 for both tasks.

**Table 4:** Downstream parser-sensitivity analysis of ACS using different Spanish dependency parsing backends.^a^

Parser	Precision	Recall	F1 score
**Trigger extraction**			
spaCy	94.72	95.14	94.93
Stanza	94.57	95.25	94.90
UDPipe	94.50	95.12	94.81
**Argument extraction**			
spaCy	92.84	89.80	91.30
Stanza	92.44	89.16	90.77
UDPipe	92.42	89.14	90.75

a All scores are reported as percentages (%).

Moreover, we measured the average processing time per document on the test set by comparing the standard sentence-level setting with the ACS-enabled setting. The average processing time increased from 293.79 to 390.97 ms/document. Breaking down the runtime, preprocessing increased from 94.77 to 189.94 ms/document, whereas prediction and post-processing changed only marginally, from 199.02 to 201.03 ms/document. These results indicate that the additional overhead introduced by ACS is concentrated in preprocessing.

##### ACS success and fallback behaviour

Using spaCy parser, ACS was applied to all 7377 sentence-level inputs in the test set. Of these, 372 sentences (5.04%) were successfully split into clause-level contexts. These successful splits correspond to 9.60% of the 3873 sentences that reached the syntactic validation step after punctuation-based splitting. These results suggest that ACS behaves conservatively, selectively decomposing structurally suitable sentences while preserving broader context elsewhere.

#### Knowledge base analysis

In this section, we characterize the constructed domain dictionaries and quantify the effect of dictionary-based knowledge infusion on extraction performance. Because trigger mentions denote substance references, whereas arguments encode open-ended attributes, we employ different construction strategies, leading to markedly different vocabulary profiles (Table 5). We therefore discuss triggers and arguments separately and relate intrinsic coverage patterns to the corresponding ablation results (Table 6). Finally, we assess the semantic reliability of the LLM-generated aliases using LLM-as-a-judge evaluation and manual spot checks of randomly sampled subsets, reporting invalid-alias estimates separately for trigger and argument expansion (Table 7).

**Table 5:** Vocabulary statistics of the domain dictionaries across construction stages.^a^

Source	Raw unique	After translation and filtering	After alias generation
**Internal dictionaries**			
ToxHabits (trigger)	767	–	–
ToxHabits (argument)	2 893	–	15 297
**External dictionaries**			
DrugBank (formal)	51 211	11 684	12 632
DDI (formal)	1 339	1 153	1 304
RedMed (informal)	34 876	6 392	7 011
WebData (informal)	12 064	4 334	4 682

a ‘After translation and filtering’ reports trigger vocabulary after LLM-assisted cross-lingual translation and subsequent filtering; it is not applied to argument lexicons (–). ‘After alias generation’ reports vocabulary after expanding each lexicon with LLM-generated surface-form variants.

**Table 6:** Ablation study of the knowledge infusion scheme.^a^

Configuration	Precision	Recall	F1 score
**Trigger extraction**			
(I) Baseline (no infusion)	93.56	94.02	93.79
(II) Internal trigger dictionary only	93.76	94.16	93.96
(III) Internal and external dictionary (no aliases)	94.62	94.88	94.75
(IV) Full dictionary (with aliases)	94.72	95.14	94.93
**Argument extraction**			
(I) Baseline (no infusion)	92.42	88.41	90.37
(II) Internal argument dictionary only	92.83	88.74	90.74
(III) LLM-expanded internal dictionary	92.85	89.80	91.30

a For argument extraction, we apply LLM-based expansion of the internal dictionary rather than external resources. All scores are reported as percentages (%).

**Table 7:** Hallucination rates estimated using two LLM judges and manual human spot-checking.

	Trigger		
Assessment source	Translation (%)	Alias generation (%)	Argument alias generation (%)
DeepSeek-V4-Flash	0.02	1.74	5.47
GPT-5.4-mini	0.43	5.49	6.55
Human spot-check	0.11	2.05	3.12

**Figure 7:**
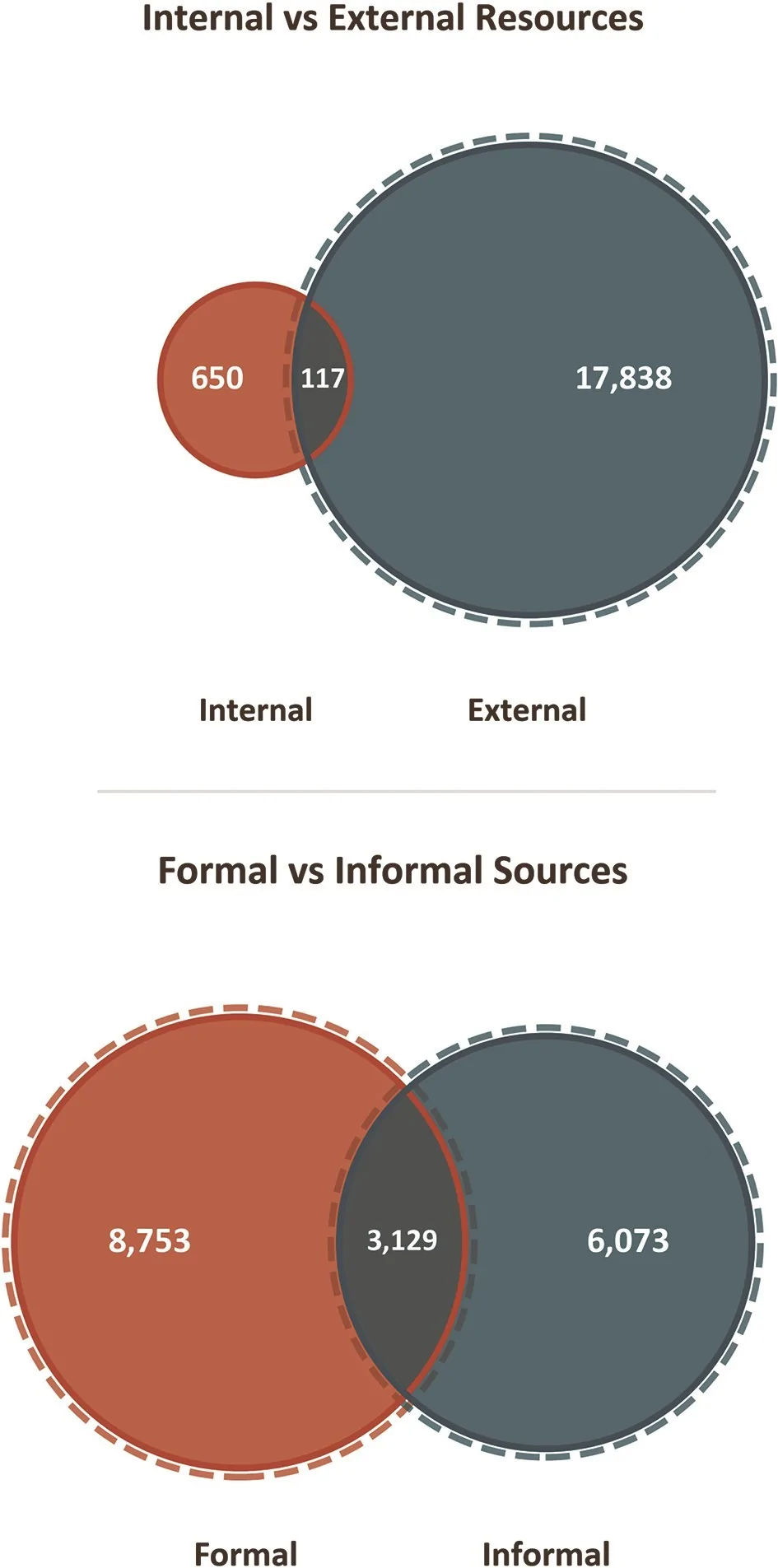
Venn diagrams illustrating the overlap of trigger vocabularies across resource categories. Numbers report base vocabularies after cross-lingual translation and filtering (before alias generation); overlaps are computed on these base sets. Solid circles indicate no alias expansion, whereas dashed outlines indicate alias-generated variants. Top: overlap between internal and external resources. Bottom: overlap between formal (DrugBank, DDI) and informal (RedMed, WebData) sources.

**Figure 8:**
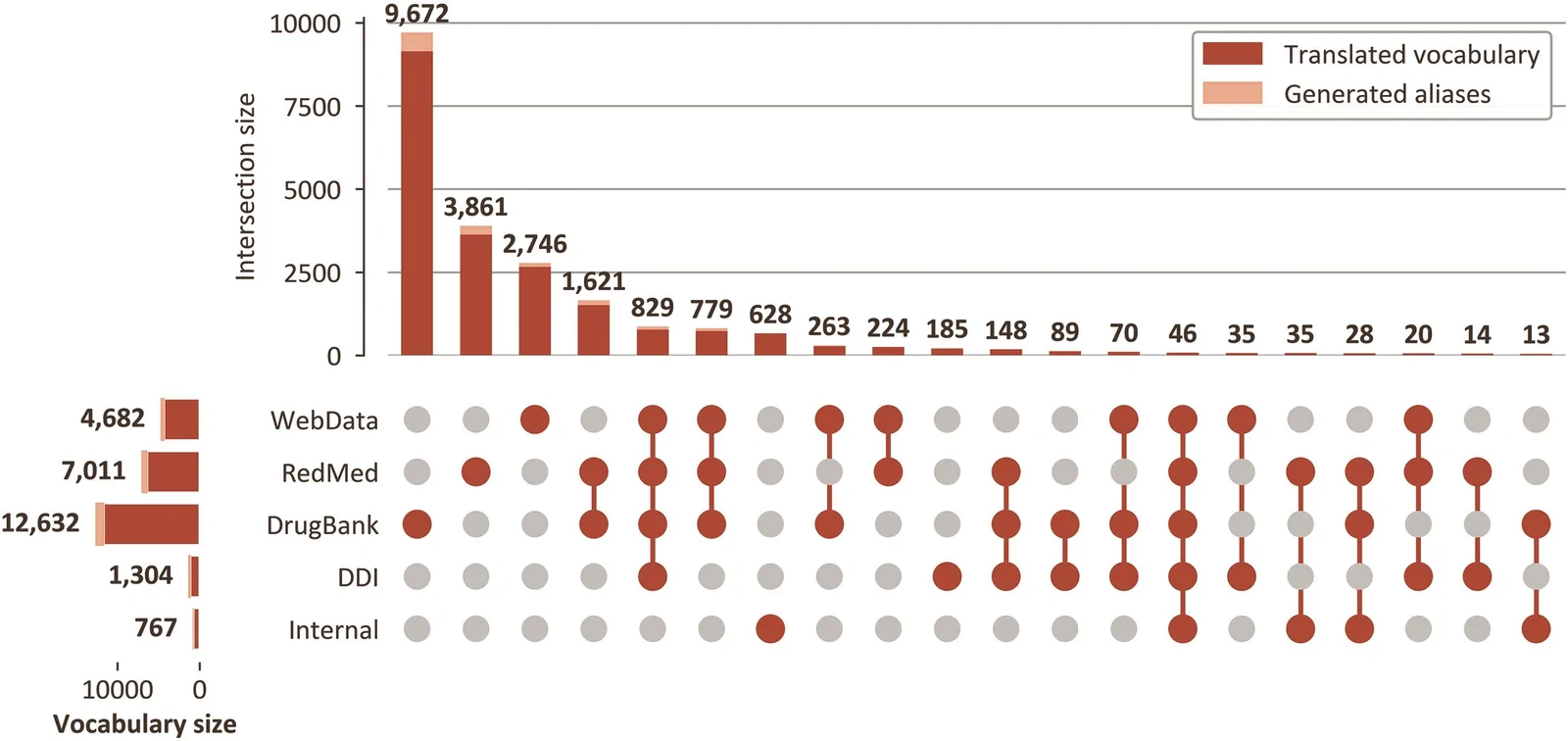
UpSet plot analysing the trigger vocabulary composition across five data sources after the LLM enrichment step in dictionary construction. (1) The horizontal bars on the left represent the total vocabulary size of each dataset, comprising both translated vocabulary and generated aliases. (2) Similarly, the upper vertical bars display the aggregate count for each intersection, stacked to show the total size. (3) The matrix below visualizes the specific combinations of intersecting datasets; connected dots indicate shared vocabulary among linked sources, while solitary dots represent unique terms exclusive to a single source.

For *triggers*, external resources are essential to mitigate lexical sparsity in the training set. As shown in Fig. 7, the internal vocabulary contains 767 unique terms, of which only 117 overlap with external resources, while 17 838 additional terms are contributed externally. This means that training-only supervision exposes the model to merely *4%* of the combined domain lexicon, motivating explicit lexical enrichment. Decomposing external sources into formal (DrugBank, DDI) and informal (RedMed, WebData) resources reveals complementary coverage. Informal sources add 6073 unique terms, capturing colloquial and non-standard mentions absent from standardized ontologies. Intersection analysis (Fig. 8) further shows that no single resource is sufficient: DrugBank contributes the largest unique subset (9672 terms), while RedMed and WebData provide substantial exclusive coverage. These coverage patterns align with the ablation results (Table 6). Internal-only infusion yields marginal improvement, whereas incorporating external resources increases performance to *94.75%* F1 and boosts recall to *94.88%*. Alias generation provides an additional consistent gain, raising F1 to *94.93%* through further recall improvement (*95.14%*).

For *arguments*, external knowledge bases provide limited support due to the absence of specialized attribute-level resources. Unlike triggers, which form a relatively bounded set, arguments are expressed through open-ended language patterns with high surface variability. We therefore expand the argument dictionary from internal seeds using controlled LLM-based generation. As shown in Table 5, this process enlarges the vocabulary from 2893 to 15 297 terms, representing substantial phrasal enrichment. This expansion is reflected in the ablation results (Table 6). The static internal dictionary yields only marginal gains, whereas LLM-based expansion significantly improves performance, increasing recall from *88.74%* to *89.80%* and F1 to *91.30%*.

To quantify lexical noise introduced during LLM-assisted dictionary construction, we defined a hallucinated translation as a generated Spanish term that failed to preserve the referent of its English source term. For alias generation, a trigger alias was considered invalid if it denoted a different substance or substance use concept, whereas an argument alias was considered invalid if it was not a plausible span for its inherited argument category. We assessed the complete candidate inventories using two independent LLM models, DeepSeek-V4-Flash (DeepSeek API documentation: https://api-docs.deepseek.com/, accessed 1 July 2026) and GPT-5.4-mini (OpenAI model documentation: https://developers.openai.com/api/docs/models/gpt-5.4-mini, accessed 1 July 2026), complemented by manual spot checks of random samples comprising 950 trigger and 750 argument candidates, corresponding to ∼5% of each dictionary vocabulary (Table 7). Translation hallucination rates were low, ranging from *0.02%* to *0.43%* across automated assessments, with a similarly low manual estimate of *0.11%*. This likely reflects the constrained prompting setup and the short source–target mappings, in which most English terms were mapped to one- or two-token Spanish forms with limited generative latitude. In contrast, trigger alias generation produced higher and more variable invalid-alias rates, indicating greater opportunity for semantic drift when multiple surface variants are generated. Manual inspection of disagreements between the two LLM judges indicated that GPT-5.4-mini tended to apply a stricter entity-identity criterion than DeepSeek-V4-Flash, more often rejecting indirect substance associations, overly generic forms, and non-standard lexical variants; however, some rejected cases were still clinically plausible, suggesting occasional over-rejection of valid aliases. For argument aliases, automated invalid-alias estimates (5.47–*6.55%*) exceeded the manual spot-check estimate (*3.12%*), indicating more conservative category boundaries in the LLM-based verification. The invalid-alias rate for arguments may also reflect the expansion design, where each argument seed generated a relatively large set of contextual variants, increasing the opportunity for semantic drift or category-boundary ambiguity.

#### Data augmentation analysis

**Figure 9:**
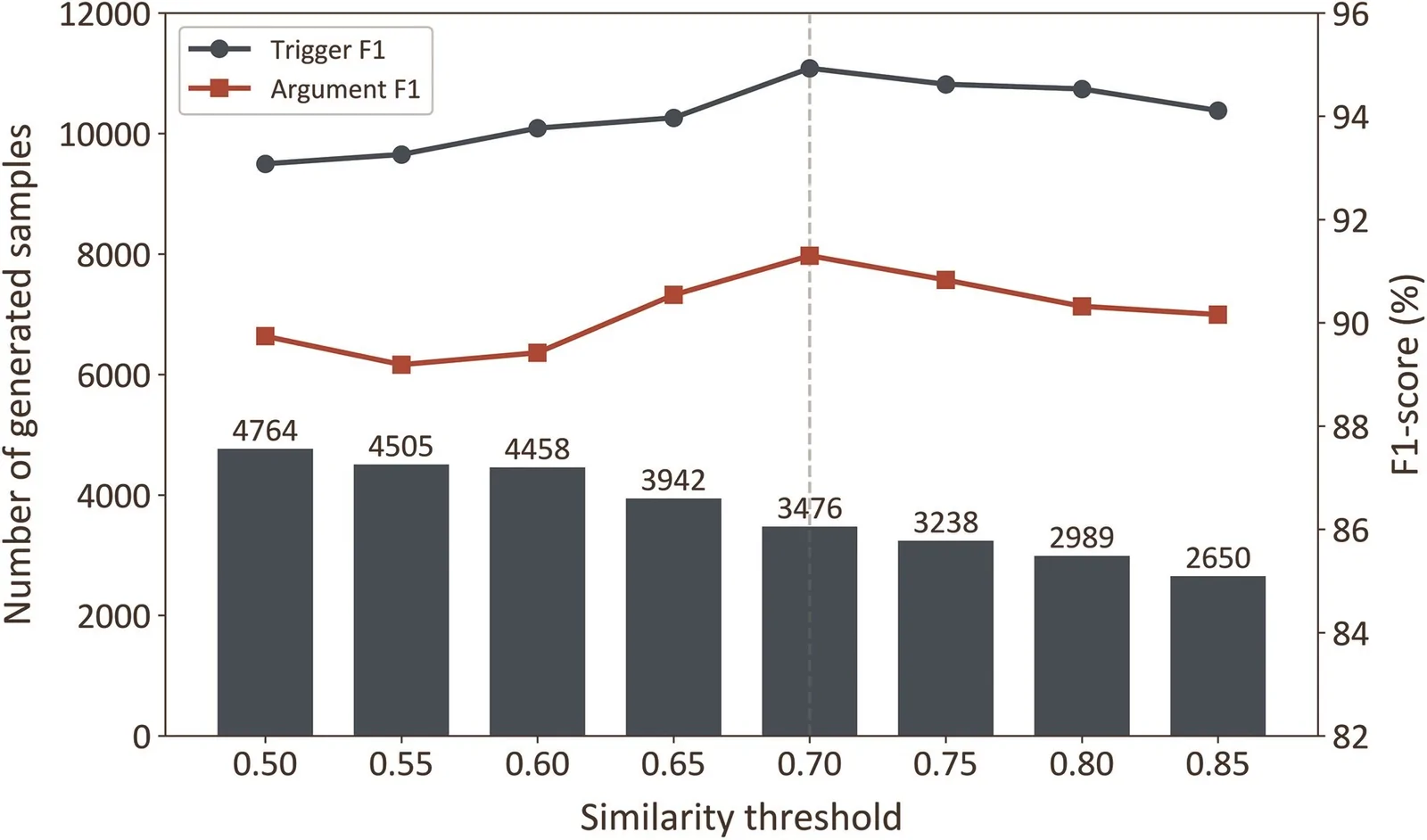
Effect of the similarity threshold on context-aware data augmentation. Bars indicate the number of generated training samples under each threshold after the first round of self-training, while lines show the F1 scores for trigger and argument extraction on the development set. All scores are reported as percentage (%).

To further analyse the effect of the semantic similarity threshold in our proposed context-aware data augmentation module, we report results under different threshold values after the first round of self-training in Fig. 9. During augmentation, we generate samples at a fixed one-to-one ratio with the original training data and conduct experiments with similarity thresholds ranging from 0.5 to 0.85.

The effect of the similarity threshold becomes evident after the first self-training round. As the threshold increases, the number of retained augmented samples decreases from 4764 at 0.50 to 2650 at 0.85, reflecting stricter semantic filtering. Performance improves up to a threshold of 0.70, with trigger and argument F1 reaching 94.93 and 91.30, respectively. These results suggest that moderate constraints effectively balance noise reduction and useful diversity.

Beyond 0.7, performance declines as stricter constraints excessively reduce augmented data. At 0.85, trigger and argument F1 drop to 94.11 and 90.16, respectively. Overall, a threshold of 0.7 achieves the best balance between data quantity and semantic quality, retaining 3476 samples and yielding the highest F1 scores.

#### Model generalization analysis

To evaluate the general applicability of our proposed framework beyond the primary dataset, we conduct cross-dataset experiments on the DreamDrug corpus [62]—an annotated NER dataset for extracting drugs in darknet markets. The dataset consists of >3500 user-generated item listings from darknet markets, with nearly 15 000 annotated mentions of the Drug entity type. Using this corpus, we adopt the same core architecture and learning strategies without introducing any dataset-specific modifications. We follow the original DreamDrug evaluation protocol and metrics to ensure a fair and consistent comparison with previously reported state-of-the-art approaches. Performance is evaluated on both the development and testing sets and compared against baselines under different domain-adaptive pretraining (DAPT) settings.

Table 8 presents a comparison between our framework and strong transformer-based baselines under different domain-adaptive pretraining settings. Among the reported approaches, RoBERTa with domain-adaptive pretraining achieves competitive precision but relatively lower recall. In contrast, our model consistently attains the highest recall and the best F1 score on both the development and testing sets. Specifically, without any domain-adaptive pretraining, our framework achieves *84.08%* F1 on the development set and *85.01%* on the test set, outperforming the strongest reported RoBERTa-based baseline (*83.86%* F1 on the test set). These results demonstrate that our method remains competitive with, and surpasses, existing state-of-the-art models on DreamDrug. Notably, this result is obtained using the same core architecture and learning strategy developed for our primary task, without introducing architectural modifications specifically for DreamDrug. Overall, the findings indicate that our architectural design generalizes effectively to a distinct and noisy dataset, achieving strong performance without dataset-specific adaptation

**Table 8:** Performance comparison on the DreamDrug dataset under different domain-adaptive pretraining (DAPT) settings.^a^

	Development set				Testing set			
Model	DAPT	Precision	Recall	F1 score	DAPT	Precision	Recall	F1 score
BERT	All	85.71	77.94	81.64	–	–	–	–
RoBERTa	None	81.13	80.56	80.84	–	–	–	–
RoBERTa	Wiki	84.86	79.62	82.16	Wiki	**84.83**	82.92	83.86
RoBERTa	All	**86.63**	76.43	81.21	–	–	–	–
Our proposed model	None	81.63	**86.68**	**84.08**	None	84.21	**85.83**	**85.01**

a Our model is trained without DAPT and achieves the best recall and F1 score on both the development and testing sets. Bold values indicate the highest reported score for each metric within each data split. All scores are reported as percentages (%).

## Discussion

Table 9 presents representative error cases that illustrate both the shared limitations of NOWJ/I-NOWJ and the improvements and trade-offs introduced by our proposed approach.

**Table 9:** Examples of errors in ToxNER (trigger extraction) and ToxUse (argument extraction) caused by NOWJ and I-NOWJ on the ToxHabits corpus.^a^

No.	Document ID	Sentence	I-NOWJ	NOWJ	Gold	TP/FP/FN ^b^	Cause of error
1	S0211-69952010 000500016-1	Varón de 20 años, fumador y ex-consumidor de cocaína y cannabis ... *(20-year-old man, smoker and former user of cocaine and cannabis...)*	Fumador [Tobacco]	Fumador [Tobacco]	Fumador [Alcohol]	1 FN, 1 FP	Labelling error in the gold standard.
2	caso_clinico_ radiologia924	Ex tabaquista (TBQ), dejó hace 5 años, fumó durante 60 años en promedio 15 cigarrillos por día (80 p/y). *(Former smoker (TBQ), quit 5 years ago, smoked for 60 years, averaging 15 cigarettes per day (80 p/y).)*	15 cigarrillos [Amount]	15 cigarrillos [Amount]	promedio 15 cigarrillos [Amount]	1 FN, 1 FP	Boundary mismatch arising from multi-word expressions.
3	caso_clinico_ radiologia924	Ex tabaquista (TBQ), dejó hace 5 años, fumó durante 60 años en promedio 15 cigarrillos por día (80 p/y). *(Former smoker (TBQ), quit 5 years ago, smoked for 60 years, averaging 15 cigarettes per day (80 p/y).)*	Tabaquista [Tobacco]	Tabaquista [Tobacco]	Tabaquista (TBQ) [Tobacco]	1 FN, 1 FP	Boundary errors by trigger abbreviation.
4	caso_clinico_ urgencias100	Actualmente niega consumo desde hace 5 años. *(He currently denies having used for the past 5 years.)*	–	–	Consumo [Drug]	1 FN	Entity omission caused by the trigger’s high level of abstraction.
5	cc_habitos_ toxicos460	El análisis toxicológico fue positivo para cocaína, benzodiacepinas, metoclopramida y posibles opiáceos. *(The toxicological analysis was positive for cocaine, benzodiazepines, metoclopramide and possible opiates.)*	Metoclopramide [Drug]	–	Metoclopramide [Drug]	1 TP	Rare trigger extraction by knowledge-enhanced module.
6	cc_habitos_ toxicos922	Pero después volvió a fumar algún porro y tomar alguna droga... *(But later he started smoking some joints again and taking some drugs ...)*	Porro [Type]	–	Porro [Type]	1 TP	Rare argument extraction by knowledge-enhanced module.
7	cc_habitos_ toxicos756	A este paciente nunca le gustó la bebida alcohólica, pero comenzó a consumirla después de un accidente... y él resultó lesionado... *(This patient never liked alcoholic beverages, but began consuming them after an accident ... and he was injured ...)*	Bebida alcohólica [Alcohol]	–	Bebida alcohólica [Alcohol]	1 TP	Correct prediction enabled by selective context focus.
8	casos_clinicos_ habtox20	A los 22 años el consumo de alcohol era ya diario y el de cannabis se hizo más habitual al tener más poder adquisitivo, y la cantidad y la frecuencia de consumo de ambos tóxicos aumentó con el paso de los años. *(By the age of 22, alcohol consumption had already become daily, and cannabis use became more frequent as purchasing power increased; over the years, both the amount and the frequency of use of these substances increased.)*	Más habitual [Frequency]	–	Más habitual [Frequency]	1 TP	Model enhanced by selective context focus.
9	cc_habitos_ toxicos607	Estos datos confirman la gravedad potencial del consumo de GHB y de sus precursores, que puede expresarse tanto en forma de sobredosis como de síndrome de abstinencia. *(These data confirm the potential severity of GHB use and its precursors, which may manifest either as overdose or as withdrawal syndrome.)*	GHB [Drug]	–	–	1 FP	False positive arising from domain-specific bias.
10	cc_habitos_ toxicos9	El varón más joven consumió todo el preparado, mientras que el otro consumió 2/3. *(The younger man consumed all of the preparation, while the other consumed 2/3.)*	2/3 [Amount]	–	–	1 FP	False positive caused by distal-context pruning.
11	cc_habitos_ toxicos232	Al alta: Bupremorfina/naloxona 12 mg/día, se mantiene estable 10 meses hasta que en Unidad del Dolor (UD) se prescribe Fentanilo oral. *(At discharge: Buprenorphine/naloxone 12 mg/day; he remained stable for 10 months until oral fentanyl was prescribed at the Pain Unit (PU).)*	–	Buprenorfina/ naloxona [Drug]	Buprenorfina/ naloxona [Drug]	1 FN	False negative caused by distal-context pruning.

a Rows 1–4 show inherent limitations that neither I-NOWJ nor NOWJ can resolve; rows 5–8 highlight cases where I-NOWJ outperforms NOWJ; and rows 9–11 illustrate the trade-offs of I-NOWJ introducing new errors compared to NOWJ.

b *TP: Correctly predicted positive cases. FP: Incorrectly predicted positive cases. FN: Missed positive cases*.

Regarding common limitations, both models are affected by annotation inconsistencies and the inherent complexity of clinical substance-related language (see examples 1–4). For instance, *fumador* (‘smoker’) is labelled as Alcohol, although it more naturally corresponds to Tobacco, creating an apparent error despite a semantically reasonable prediction. Compact forms such as *tabaquista (TBQ)* (‘smoker’) also complicate exact boundary extraction when full forms and abbreviations occur within the same span.

Errors further arise from vague or underspecified contexts. In *Actualmente niega consumo desde hace 5 años* (‘He currently denies having used for the past 5 years’), the trigger consumo lacks an explicit substance reference, making reliable classification difficult even for a context-aware model.

Beyond these shared limitations, I-NOWJ shows clear improvement over NOWJ by leveraging external knowledge and ACS (see Examples 5–8). I-NOWJ successfully extracts rare pharmaceutical triggers such as *metoclopramide*, which are missed by NOWJ due to limited training exposure. The model also correctly identifies informal expressions like *porro* (‘joint’), demonstrating the benefit of knowledge-enhanced representations for capturing non-standard mentions. Moreover, ACS improves robustness in long and noisy narratives. In Examples 7 and 8, where mentions such as *bebida alcohólica* (‘alcoholic beverages’) and *más habitual* (‘more frequent’) are surrounded by irrelevant information, NOWJ produces incorrect predictions, whereas I-NOWJ selectively attends to relevant cues and yields correct predictions.

Nevertheless, these gains introduce trade-offs. Integrating external knowledge can increase sensitivity to insufficient context, leading to errors when a lexically matched term appears without adequate supporting evidence. Not all matched terms function as true substance triggers; some serve only descriptive roles, resulting in false positives under ambiguous conditions (see Example 9). In addition, decomposing long sentences into smaller segments may remove crucial distant context, adversely affecting predictions (Examples 10 and 11).

Overall, despite these limitations, Table 9 shows that I-NOWJ remains more robust and effective than NOWJ in handling domain-specific substance expressions and complex, context-dependent clinical narratives.

While I-NOWJ improves robustness over the baseline, several limitations remain. Its effectiveness partly relies on the availability and coverage of external domain knowledge, which may vary across datasets. Future work could explore dynamic knowledge expansion to enhance generalization. Although ACS mitigates noise in lengthy narratives, the current framework does not model discourse-level dependencies. Incorporating document-level context or temporal reasoning may improve the handling of longitudinal substance use descriptions. Finally, as clinical and informal language evolves, more flexible strategies for adapting to emerging terminology without extensive retraining are needed. Addressing these challenges would further strengthen the applicability of I-NOWJ in real-world clinical settings.

## Conclusion

In this paper, we introduced I-NOWJ, a unified framework for extracting substance use information from Spanish clinical narratives under heterogeneous conditions. By integrating ACS, knowledge-guided learning, joint trigger–argument optimization, and data augmentation, the proposed approach effectively addresses lexical scarcity and contextual variability, consistently outperforming existing systems and improving upon our previous shared task submission. Beyond overall performance gains, our analysis reveals important characteristics of substance use extraction. While trigger detection remains relatively robust under strict boundary evaluation, argument extraction shows a larger gap between exact-match and overlap-based metrics, suggesting that boundary precision remains a key challenge despite correct semantic localization. Ablation results further demonstrate that effective extraction depends on dynamic context selection rather than fixed contextual scopes. The adaptive strategy reduces irrelevant noise while preserving essential structural cues, improving robustness in complex clinical texts. Combined with multi-source domain knowledge integration and controlled data augmentation, the framework enhances generalization in low-resource settings. Although the current study focuses on Spanish ToxHabits data, future work will explore more flexible context selection mechanisms, improved span refinement for argument boundaries, and broader cross-lingual generalization. We believe that the proposed framework provides a solid foundation for advancing substance use information extraction in low-resource clinical scenarios.

## Data Availability

The data underlying this article are all available: The BioCreative IX ToxHabits dataset is available from the BioCreative IX ToxHabits organizers; access information is provided at: https://zenodo.org/records/15538314 DrugBank Open Data (version 5.1.13) is publicly available at: https://go.drugbank.com/releases/5-1-13 The DDI corpus is publicly available at: https://zenodo.org/records/10560167 The RedMed lexicon is publicly available at: https://github.com/alavertu/redmed The FDA Drugs@FDA data files are available at: https://www.fda.gov/drugs/drug-approvals-and-databases/ drugsfda-data-files The Kaggle Illicit Drugs dataset (version 1) is available at: https://www.kaggle.com/datasets/willianoliveiragibin/ illicit-drugs/versions/1 The Wikipedia list of slang names for cannabis is available at: https://en.wikipedia.org/wiki/List_of_slang_names_for _cannabis The Wikipedia list of alcoholic drinks is available at: https://en.wikipedia.org/wiki/List_of_alcoholic_drinks The DreamDrug corpus is available upon request from the dataset authors (DreamDrugDataset@gmail.com). Our source code and constructed dictionaries are available at: https://github.com/candleMind/toxhabit
